# Multi-Cue Kinetic Model with Non-Local Sensing for Cell Migration on a Fiber Network with Chemotaxis

**DOI:** 10.1007/s11538-021-00978-1

**Published:** 2022-02-12

**Authors:** Martina Conte, Nadia Loy

**Affiliations:** Department of Mathematical Sciences, “G. L. Lagrange”, Politecnico di Torino, Corso Duca degli Abruzzi 24, 10129 Torino, Italy

**Keywords:** Non-local kinetic equations, Multi-cue cell migration, Multiscale modeling, Hydrodynamic limit, Contact guidance, Chemotaxis, 35Q20, 35Q92, 92B05, 45K05, 92C17

## Abstract

Cells perform directed motion in response to external stimuli that they detect by sensing the environment with their membrane protrusions. Precisely, several biochemical and biophysical cues give rise to tactic migration in the direction of their specific targets. Thus, this defines a multi-cue environment in which cells have to sort and combine different, and potentially competitive, stimuli. We propose a non-local kinetic model for cell migration in which cell polarization is influenced simultaneously by two external factors: contact guidance and chemotaxis. We propose two different sensing strategies, and we analyze the two resulting transport kinetic models by recovering the appropriate macroscopic limit in different regimes, in order to observe how the cell size, with respect to the variation of both external fields, influences the overall behavior. This analysis shows the importance of dealing with hyperbolic models, rather than drift-diffusion ones. Moreover, we numerically integrate the kinetic transport equations in a two-dimensional setting in order to investigate qualitatively various scenarios. Finally, we show how our setting is able to reproduce some experimental results concerning the influence of topographical and chemical cues in directing cell motility.

**Supplementary Information:**

The online version contains supplementary material available at 10.1007/s11538-021-00978-1.

## Introduction

Cell migration is a fundamental mechanism in a huge variety of processes, such as wound healing, angiogenesis, tumor stroma formation, and metastasis. During these processes, cells sense the environment and respond to external stimuli orienting their direction of motion toward specific targets. This mechanism is referred to as *taxis*, and it results in the persistent migration in a certain preferential direction. The guidance cues leading to directed migration may be biochemical or biophysical. One example of a biochemical cue is the concentration of soluble molecules in the extracellular space. This cue gives rise to *chemotaxis*, which is considered a *mono-directional* stimulus. Other cues generating mono-directional stimuli include electric fields (*electrotaxis*, or *galvanotaxis*), light signals (*phototaxis*), bound ligands to the substratum (*haptotaxis*), or the extracellular matrix (ECM) stiffness (*durotaxis*) (Lara and Schneider 2013). Precisely, ECM stiffness can be counted as a biophysical cue, as well as the collagen fiber alignment. In particular, the latter is shown to stimulate *contact guidance* (Friedl and Brocker 2000; Friedl 2004), i.e., the tendency of cells to migrate by crawling on the fibers and following the directions imposed by them. Contact guidance is a *bi-directional* cue. In fact, if the fibers are not polarized, there is no preferential sense of motion along them.

In many pathological and physiological processes, there are several directional cues inducing different simultaneous stimuli. While the cell’s response to each of them has been largely studied, the cell’s response to a multi-cue environment is much less understood. Some studies have shown how there can be competition or cooperation between these different stimuli. Thus, the fundamental issue concerns the way cells rank, integrate, or hierarchize them, especially when these stimuli are competing (e.g., when they are not co-aligned) (Rajnicek et al. 2007). Therefore, with the present work we propose a kinetic model aimed at analyzing cell behavior in response to two different stimuli. We study the way the simultaneous sensing of two cues—chemotaxis and contact guidance—influences the choice of the cell migratory direction. We take into account non-local sensing of both cues, since cells extend their protrusions in order to sense the environmental stimuli.

### Biological Background

The coexistence of chemotaxis and contact guidance happens in vivo in a variety of situations, like wound healing or cancer progression. For example, during wound healing, fibroblasts migrate efficiently along collagen or fibronectin fibers in connective tissues. In tumor spread and metastasis formation, cancer cells follow the aligned fibers at the tumor-stroma interface and, thus, are facilitated to reach blood and lymphatic vessels (Steeg 2016; Provenzano et al. 2006, 2009). In both cases, chemotactic gradients have been shown to accelerate and enhance these processes (Lara and Schneider 2013; Bromberek et al. 2002). Another important issue concerns the design of platforms for controlling multiple directional cues and, in particular, soluble factors and aligned fibers. In fact, there are not many experimental studies that look at the combined effect of chemotaxis and contact guidance (Lara and Schneider 2013). In one of the first works on this topic (Wilkinson and Lackie 1983), the authors analyze contact guidance of neutrophil leukocytes on fibrils of collagen, showing a more efficient migration in the fiber direction, instead of in the perpendicular one. They also observe that, in the presence of a chemoattractant, there is cooperation or competition between the cues depending on their relative orientations. In the work by Bromberek et al. (2002), the enhancement of the alignment along the fibers is observed in presence of a co-aligned chemoattractant, while, in Maheshwari et al. (1999), the authors study the effects of different fibronectin densities and growth factor (EGF) concentrations on the quantitative regulation of random cell migration. An interesting 2D platform, allowing to study contact guidance and chemotaxis, was proposed by Sundararaghavan et al. (2013). Here, the authors demonstrate an additive effect of chemical gradients and fiber alignment by measuring the persistence time and a stronger dominance of contact guidance when the chemotactic gradient is aligned perpendicularly to the fibers. Thus, as for multiple directional cues different scenarios may happen, a deep understanding of cell migrational responses is a key step for the comprehension of both physiological and pathological processes.

### Mathematical Models for Directed Cell Migration

There is a huge variety of mathematical models concerning different aspects of directed cell migration. They range from microscopic (also called individual-based) models, describing migration at the cell level, up to macroscopic ones, concerning collective cell migration at a tissue level.

Among individual-based models, there are many examples regarding separately chemotaxis (e.g., see Di Costanzo et al. 2020; Giniūnaitė et al. 2019 and references therein) and migration on the ECM (Colombi et al. 2017; Scianna et al. 2013; Schlüter et al. 2012). Concerning macroscopic settings, the famous *Keller–Segel model* is one of the first examples of a drift-diffusion system postulated at the macroscopic level for the study of the chemotactic effects (Keller and Segel 1970). Then, many efforts were made in order to encompass some defects of this setting, as well as for deriving it from lower scale models (e.g., see Painter 2019; Hillen and Painter 2008; Othmer and Hillen 2002; Othmer and Stevens 2001; Bellomo et al. 2015 and references therein). Between microscopic and macroscopic models, the mesoscopic settings represent an intermediate scale, which includes microscopic dynamics and describes the statistical distribution of the individuals. As in the case of kinetic theory, these models allow to recover the appropriate macroscopic regime, which inherits some details of the microscopic dynamics and, thus, gives more significance to some of the derived parameters (e.g., see Othmer and Hillen 2002; Colombi et al. 2015; Chalub et al. 2004; Eftimie 2012 and references therein). The two major models describing contact guidance at the mesoscopic level were proposed by Dickinson (2000) and Hillen (2006), and both models are local in the physical space.

Although the wide literature concerning single-cue models, not many models exist for multiple cues, especially settings describing in details the process of cell sensing. Among the few examples of multiple-cue analysis, we recall the work proposed by Painter et al. (2000), where a macroscopic drift-diffusion model is derived from a space jump process describing the response to multiple chemicals. A review of macroscopic PDEs including multiple-taxis has been recently proposed by Kolbe et al. (2021). Looking specifically at the combination of chemotaxis and contact guidance, in Wagle and Tranquillo (2000) one of the first models describing microscopic dynamics is proposed, while, in Azimzade et al. (2019), a microscopic double-cue stochastic model was introduced to analyze cell migration and classify tumor associated collagen signatures (TACS). We also recall the kinetic models for cell–cell interactions on a fiber network in presence of a tactic cue presented by Chauviere et al. (2007). Recently, an extension of the M5 model (Hillen 2006) was extended in order to include chemotaxis, with the aim of showing network formation in the presence of a chemoattractant (Thiessen and Hillen 2021). Non-locality in the description of cell sensing over a finite neighborhood and in presence of two external cues was firstly introduced by Loy and Preziosi (2020, 2020). This non-local kinetic model takes into account the dependence of the cell sensing on the cell size (e.g., on the maximum cell protrusion length) and includes two cues, one affecting cell direction, while another influencing cell speed.

### Outline of the Paper

Due to the lack of mathematical models looking at the interplay between chemotaxis and contact guidance from a non-local perspective and the biological relevance of this topic, in this paper we develop a non-local kinetic model describing the combined effect of these two directional cues on the choice of the direction of cell migration. We propose a transport equation implementing a velocity-jump process in which the transition probability describing the choice of the new velocity takes into account the non-local sensing of the fiber network and a chemoattractant. In particular, we propose two possible sensing strategies: in the first case, cells can measure the guidance cues independently and weight them differently; in the second case, cells measure simultaneously the two cues and weight them equally. To the best of our knowledge, this is the first time that a non-local sensing of the fibers distribution defined at a mesoscopic level is considered and is coupled with a non-local sensed chemoattractant. As the kinetic approach allows to derive macroscopic models, we will discuss how the choices of the transition probability, together with the size of the sampling volume and the characteristic variability of the two cues, determine the macroscopic behaviors. This analysis, along with the numerical simulations, will show how different regimes can lead to very different macroscopic equations and that a drift-diffusion model directly stated at the macroscopic level is not always capable of capturing this dynamics.

Specifically, in Sect. 2.1 we present some mathematical preliminaries guiding the reader toward a more clear understanding of the mathematical approach, while the novel characteristic aspects of our transport models are introduced in Sect. 2.2. Then, in Sect. 3 we derive the macroscopic models. Precisely, after the brief introduction of Sect. 3.1 about the well known techniques based on asymptotic expansions we use in the following sections, we shall analyze the possible macroscopic regimes in Sect. 3.2, in relation to both cell size and characteristic lengths of variation of the cues. In Sect. 3.3 and 3.4, we derive the macroscopic limits in the various regimes starting from the transport models implementing the two different sensing strategies. In Sect. 4, we present different numerical tests, in which we numerically integrate the kinetic transport equations in order to qualitatively investigate various scenarios in a two-dimensional setting. We highlight the main features of our approach and make some considerations about the derived macroscopic models. Finally, in Sect. 5, we present an application of our model to an experimental setting, while in Sect. 6 we shall draw some conclusions and illustrate the potential and the possible outcomes of our work.

## A Kinetic Model for Chemotaxis on a Fiber Network

### Preliminaries

The cell population is described at a mesoscopic level through the distribution density ${p = p(t,\mathbf{x}, v,\hat{\mathbf{v}})}$ that, for every time $t>0$ and position $\mathbf{x}\in \Omega \subseteq {\mathbb {R}}^d$, gives the statistical distribution of the speeds $v\in [0,V]$, where *V* is the maximal speed a cell can achieve, and of the polarization directions $\hat{\mathbf{v}}\in {\mathbb {S}}^{d-1}$, being ${\mathbb {S}}^{d-1}$ the unit sphere boundary in ${\mathbb {R}}^d$. Thus, the velocity vector will be given by $\mathbf{v}=v\hat{\mathbf{v}}$. A macroscopic description for the cell population can be classically (Cercignani 1987) recovered through the definition of moments of the distribution function *p*. Precisely, we can define the macroscopic cell density in the physical space

1 $$\begin{aligned} \rho (t,\mathbf{x}) = \int _{{\mathbb {S}}^{d-1}}\int _0^{V} p(t,\mathbf{x},v,\hat{\mathbf{v}}) \,dv\,d\hat{\mathbf{v}}\,, \end{aligned}$$

that is the expected mass in $(t,\mathbf{x})$. The mass mean velocity that is given by the average of the microscopic velocities of the normalized population $p/\rho$ can be defined as follows

$$\begin{aligned} \mathbf{U}(t,\mathbf{x})=\dfrac{1}{\rho (t,\mathbf{x})}\int _{{\mathbb {S}}^{d-1}}\int _0^{V} \mathbf{v}\, p(t,\mathbf{x},v,\hat{\mathbf{v}}) \,dv\,d\hat{\mathbf{v}}\,. \end{aligned}$$

As a consequence, the first moment of *p*

2 $$\begin{aligned} \rho (t,\mathbf{x})\mathbf{U}(t,\mathbf{x})=\int _{{\mathbb {S}}^{d-1}}\int _0^{V} \mathbf{v}\, p(t,\mathbf{x},v,\hat{\mathbf{v}}) \,dv\,d\hat{\mathbf{v}}\, \end{aligned}$$

may be seen as the mass flow. Concerning higher-order moments, we shall consider

3 $$\begin{aligned} {\mathbb {D}}(t,\mathbf{x})=\int _{{\mathbb {S}}^{d-1}}\int _0^{V} (\mathbf{v}-\mathbf{U})\otimes (\mathbf{v}-\mathbf{U}) \,p(t,\mathbf{x},v,\hat{\mathbf{v}}) \,dv \,d\hat{\mathbf{v}}. \end{aligned}$$

Since we are not considering hard spheres, as instead classically done in particle physics, and, in particular, cells are self-propelled entities with an inner energy, we shall not refer to (3) as the energy tensor or momentum flow. In particular, we only consider (3) to be a diffusion tensor of the population that is given by the variance–covariance matrix of the normalized population multiplied by the cell density. The latter gives information about the breadth of the distribution of the microscopic velocities with respect to the average velocity (Painter and Hillen 2018).

The mesoscopic model consists in the following transport equation for the cell distribution

4 $$\begin{aligned} \dfrac{\partial p}{\partial t}(t,\mathbf{x},v,\hat{\mathbf{v}}) + \mathbf{v}\cdot \nabla p(t,\mathbf{x},v,\hat{\mathbf{v}}) = {\mathcal {J}} [p] (t,\mathbf{x},v,\hat{\mathbf{v}}) \end{aligned}$$

where the operator $\nabla$ denotes the spatial gradient, so that the term $\mathbf{v}\cdot \nabla p$ takes into account the free particle transport. The term ${\mathcal {J}}[p](t,\mathbf{x},v,\hat{\mathbf{v}})$ is the *turning operator* that describes the scattering of the microscopic velocity in direction and speed. In the present case, the typical microscopic dynamics of the cell is the *run and tumble* (Block et al. 1983; Berg 1983) that prescribes an alternation of runs over straight lines and re-orientations. The choice of the new direction can be random or biased by the presence of external factors, which might attract or repel the cells. The run and tumble is classically modeled by a scattering of the microscopic velocity called velocity jump process (Stroock 1974), characterized by a *turning frequency* $\mu$ and a *transition probability* *T*. The latter models the probability of choosing a certain direction $\hat{\mathbf{v}}$ and speed *v* after a reorientation. For our purposes, we assume that the transition probability does not depend on the pre-reorientation velocity, as classically done in the pioneering work concerning kinetic equations for velocity jump processes (Stroock 1974; Othmer et al. 1988; Hillen 2006) and in the previous works with non-local transition probabilities Loy and Preziosi (2020, 2020). Therefore, the turning operator reads

5 $$\begin{aligned} {\mathcal {J}}[p](t,\mathbf{x},v,\hat{\mathbf{v}}) = \mu (\mathbf{x}) \, \Big ( \rho (t,\mathbf{x}) T(\mathbf{x},v,\hat{\mathbf{v}}) - p(t,\mathbf{x},v,\hat{\mathbf{v}}) \Big ) \,. \end{aligned}$$

As *T* is a conditional probability, it satisfies $\forall \mathbf{x}\, \in \, \Omega$

6 $$\begin{aligned} \int _{{\mathbb {S}}^{d-1}}\int _0^{V} T(\mathbf{x},v,\hat{\mathbf{v}}) dv d\hat{\mathbf{v}}=1. \end{aligned}$$

The mean macroscopic velocity after a tumble is given by the average of *T*

7 $$\begin{aligned} \begin{aligned} \mathbf{U}_T(\mathbf{x})&=\int _{{\mathbb {S}}^{d-1}}\int _0^{V}\mathbf{v}\, T(\mathbf{x},v,\hat{\mathbf{v}}) \,dv \,d\hat{\mathbf{v}}\end{aligned} \end{aligned}$$

and the variance–covariance matrix

8 $$\begin{aligned} \begin{aligned} {\mathbb {D}}_T(\mathbf{x})&=\int _{{\mathbb {S}}^{d-1}}\int _0^{V} T(\mathbf{x}, v,\hat{\mathbf{v}}) (\mathbf{v}-\mathbf{U}_T)\otimes (\mathbf{v}-\mathbf{U}_T) dv \, d\hat{\mathbf{v}}. \end{aligned} \end{aligned}$$

Since we are going to consider two-dimensional bounded domains without loss of cells and no cells coming in, we assume conservation of mass. Thus, we will require that boundary conditions, which must be imposed at the kinetic level, implement no-flux boundary conditions at the macroscopic level (also named biological no-flux boundary conditions) (Plaza 2019). Specifically, we consider, at the kinetic level, a particular prototype kind of no-flux conditions that are specular-reflecting boundary conditions, which means that cells are reflected with an angle of $\pi /2$ when they hit the wall, as previously done by Loy and Preziosi (2020).

### Structure of the Transition Probability

In this section, we introduce the transition probability modeling the decision process of a cell in the presence of two directional guidance cues: a fibrous ECM and a chemoattractant. We consider amoeboid cells moving by contact guidance without proteolysis, ${ i.e.}$, cells hit a fiber and move along the direction of the fiber (Wolf et al. 2003). It has been shown experimentally, for example in the case of glioma cancer cells (Johnson et al. 2009), that randomly disposed fibers imply isotropic cell diffusion, while aligned fibers cause anisotropic diffusion of the cells along the direction of the fibers. The first transport model (the M5 model) for contact guidance was proposed by Hillen (2006), further studied and developed in Painter (2008); Chauviere et al. (2007, 2007), and applied to the study of glioma in Painter and Hillen (2013); Engwer et al. (2015, 2016, 2017); Conte et al. (2020). Following Hillen (2006), we consider a distribution of fibers described as a distribution defined over the space of directions given by the unit sphere in ${\mathbb {R}}^d$,

9 $$\begin{aligned} q= q(\mathbf{x},\hat{\mathbf{v}}), \qquad \mathbf{x}\in \Omega , \quad \hat{\mathbf{v}}\in {\mathbb {S}}^{d-1} \end{aligned}$$

that satisfies

1. $$q(\mathbf{x},\hat{\mathbf{v}}) > 0, \quad \forall \mathbf{x}\in \Omega ,\,\, \hat{\mathbf{v}}\in {\mathbb {S}}^{d-1}$$
2. $$\displaystyle \int _{{\mathbb {S}}^{d-1}} q(\mathbf{x},\hat{\mathbf{v}}) \, d\hat{\mathbf{v}}=1, \quad \forall \mathbf{x}\in \Omega $$
3. $$q(\mathbf{x},\hat{\mathbf{v}})=q(\mathbf{x},-\hat{\mathbf{v}}), \quad \forall \mathbf{x}\in \Omega ,\,\, \hat{\mathbf{v}}\in {\mathbb {S}}^{d-1}$$.

The last condition means that we are considering a non-polarized network of fibers, so that cells are able to go in both senses in each direction. Being, then, $q(\mathbf{x},\hat{\mathbf{v}})$ a probability density distribution, we can define the mean direction of the fibers

10 $$\begin{aligned} \mathbf{E}_q(\mathbf{x})= \displaystyle \int _{{\mathbb {S}}^{d-1}} q(\mathbf{x},\hat{\mathbf{v}})\, \hat{\mathbf{v}}\, d\hat{\mathbf{v}}, \end{aligned}$$

and the variance–covariance matrix of *q*

11 $$\begin{aligned} {\mathbb {D}}_q(\mathbf{x})=\displaystyle \int _{{\mathbb {S}}^{d-1}} q(\mathbf{x},\hat{\mathbf{v}})\, (\hat{\mathbf{v}}-\mathbf{E}_q)\otimes (\hat{\mathbf{v}}-\mathbf{E}_q) \, d\hat{\mathbf{v}}\,, \end{aligned}$$

that is proved to be the diffusion tensor of the fibers (Hillen 2006). As we consider a non-polarized fiber network, we have that

12 $$\begin{aligned} \mathbf{E}_q(\mathbf{x})=0, \end{aligned}$$

meaning that there is no mean direction in the dynamics. When *q* is a regular probability distribution, the tensor (11) is symmetric and positive definite and, thus, it is diagonalizable. Each eigenvalue represents the diffusivity in the direction of the corresponding eigenvector. This means that, if the eigenvalues are equal, there is isotropic diffusion, while, if they are different, there is a preferential direction of motion, ${ i.e.}$, anisotropic diffusion. This allows to reproduce isotropic/anisotropic diffusion on a non-polarized fiber network (Hillen 2006; Painter 2008). Concerning chemotaxis, we consider a chemoattractant defined in the region $\Omega$ by a strictly positive definite function

13 $$\begin{aligned} {\mathcal {S}}={\mathcal {S}}(\mathbf{x}) :\Omega \longmapsto {\mathbb {R}}_+. \end{aligned}$$

For both chemotaxis and contact guidance, we assume that the sensing performed by the cells is non-local, as cells extend their protrusions, through which they sense the environment, up to several cell diameters (Berg and Purcell 1977). The maximum length *R* of a protrusion is called *sensing radius*. It has been firstly introduced by Othmer and Hillen (2002) for modeling a non-local gradient of a chemical and, then, used in a number of works for describing the sensing of macroscopic quantities (see Chen et al. 2019 for a review and references therein). In particular, in Loy and Preziosi (2020) and, later, in Loy and Preziosi (2020) it has been used for the non-local sensing of the cues affecting cell polarization and speed.

In the present work, we extend the model proposed in Loy and Preziosi (2020) dropping the effect of a cue on cell speed, which will be unbiased, and assuming a double sensing of cues that affect cell polarization. Thus, we assume that both ${\mathcal {S}}$ and *q* are non-locally sensed by a cell that, starting from its position $\mathbf{x}$, extends its protrusions in every direction $\hat{\mathbf{v}}\in {\mathbb {S}}^{d-1}$ up to the distance *R*. With respect to a local sensing, assuming a non-local sensing of the fiber network allows to reproduce a wider range of migration strategies that a cell can perform in order to cleverly move toward the chemoattractant. Therefore, we consider the quantities

$$\begin{aligned} {\mathcal {S}}(\mathbf{x}+\lambda \hat{\mathbf{v}}), \qquad q(\mathbf{x}+\lambda \hat{\mathbf{v}}, \hat{\mathbf{v}}), \qquad \forall \, \mathbf{x}\in \Omega ,\quad \forall \, \hat{\mathbf{v}}\in {\mathbb {S}}^{d-1}, \quad \lambda \le R. \end{aligned}$$

Next to the border of the domain $\Omega$, we always consider $\lambda$ such that $\mathbf{x}+\lambda \hat{\mathbf{v}}\in \Omega$. The term $q(\mathbf{x}+\lambda \hat{\mathbf{v}}, \hat{\mathbf{v}})$ takes into account the possibility of sensing in direction $\hat{\mathbf{v}}$ radial fibers starting from the position $\mathbf{x}$ and up to a distance $\lambda$. For the cell speed, we consider the probability density distribution $\psi$ of the speeds defined on the interval [0, *V*] that thus satisfies $\int _0^V \psi (v) dv =1$. Precisely, we define its mean speed, energy and variance as

$$\begin{aligned} {\bar{V}}=\displaystyle \int _0^V v \,\psi (v) \,dv, \quad D=\displaystyle \int _0^V v^2\, \psi (v)\, dv\,, \quad \sigma ^2_{\psi }=\dfrac{1}{2}(D-{\bar{V}}^2), \end{aligned}$$

respectively.

Regarding the sensing of the microenvironment, in general cells use different receptors located on their membrane. For instance, the interactions between cells and fibers are mediated by transmembrane receptors, such as the integrins, which allow cell–fiber bindings and the activation of related intracellular pathways. Instead, depending on the chemotactic cue, other receptors or transmembrane units, rather than integrins, can be recruited for the signal transduction. For example, endothelial cells use vascular endothelial growth factor receptors (VEGFR -1 -2 -3) in response to the chemotactic cue given by the vascular endothelial growth factors (VEGFs). Thus, on a first approximation, we can assume that cells respond independently to the information given by these different receptors and the choice of the new direction results from a combination of these two responses. Instead, on a second approximation, we can assume that cells mediate simultaneously the information coming from different receptors and the new direction is the result of a unique weighting process of the measured information. For these reasons, we propose two different transition probabilities to describe these two different sensing strategies: in the first model, the sensing of *q* and ${\mathcal {S}}$ are independent, while in the second model a unique sensing is performed.

In the first model, we introduce a transition probability that is the product of two different independent sensing processes

14 $$\begin{aligned} T[q,{\mathcal {S}}](\mathbf{x}, v,\hat{\mathbf{v}})=c(\mathbf{x})\int _{{\mathbb {R}}_+}\gamma _{{\scriptscriptstyle {\mathcal {S}}}}(\lambda ) {\mathcal {S}}(\mathbf{x}+\lambda \hat{\mathbf{v}})\, d\lambda \, \int _{{\mathbb {R}}_+}\gamma _q(\lambda )\,q(\mathbf{x}+\lambda \hat{\mathbf{v}},\hat{\mathbf{v}})\,d\lambda \, \psi (v)\,.\nonumber \\ \end{aligned}$$

In this case, a cell located in position $\mathbf{x}$ extends its protrusions up to a distance *R* in each direction $\hat{\mathbf{v}}$. Then, the cell measures the field ${\mathcal {S}}(\mathbf{x}+\lambda \hat{\mathbf{v}})$ with specific receptors, weighting the related information by $\gamma _{{\scriptscriptstyle {\mathcal {S}}}}$, and, independently, it measures the quantity $q(\mathbf{x}+\lambda \hat{\mathbf{v}},\hat{\mathbf{v}})$, weighting the related information by $\gamma _q$. The *sensing functions* $\gamma _{{\scriptscriptstyle {\mathcal {S}}}}\ge 0$ and $\gamma _q\ge 0$ have compact support in [0, *R*] and they may be Dirac deltas centered in *R*, if the cell only measures the guidance cues on its membrane (only on $\mathbf{x}+R\hat{\mathbf{v}}$ for every $\hat{\mathbf{v}}$), or Heaviside functions if the cell measures and gives the same weight to *q* and ${\mathcal {S}}$ from $\mathbf{x}$ to $\mathbf{x}+R\hat{\mathbf{v}}$ in each direction. Formally, from a stochastic point of view this choice describes an independence of the two phenomena and, thus, the transition probability can be seen as the product of the independent probabilities of *q* and ${\mathcal {S}}$, ${ i.e.}$, $T[q,{\mathcal {S}}]={\hat{T}}[q]\,{\hat{T}}[{\mathcal {S}}]$.

The second model prescribes a simultaneous averaging of the guidance cues ${\mathcal {S}}$ and *q*, ${ i.e.}$,

15 $$\begin{aligned} T[q,{\mathcal {S}}](\mathbf{x}, v,\hat{\mathbf{v}})=c(\mathbf{x})\int _{{\mathbb {R}}_+}\gamma (\lambda ) {\mathcal {S}}(\mathbf{x}+\lambda \hat{\mathbf{v}})\,q(\mathbf{x}+\lambda \hat{\mathbf{v}},\hat{\mathbf{v}})d\lambda \, \psi (v)\,. \end{aligned}$$

This transition probability describes a cell in position $\mathbf{x}$ that extends its protrusions and measures in each direction $\hat{\mathbf{v}}$ the two quantities ${\mathcal {S}}(\mathbf{x}+\lambda \hat{\mathbf{v}})$ and $q(\mathbf{x}+\lambda \hat{\mathbf{v}})$, weighting both simultaneously with the same sensing function $\gamma \ge 0$. Formally, as the two sensing are not independent, the transition probability cannot be factorized.

In (14) and (15), $c(\mathbf{x})$ is a normalization coefficient.

We refer to the transport model (4)-(5) with (14) as *non-local independent sensing model*, in which the cell averages the two cues independently according to two different sensing functions $\gamma _q$, $\gamma _{{\scriptscriptstyle {\mathcal {S}}}}$. On the other hand, the transport model (4)-(5) with (15) is defined as *non-local dependent sensing model*, describing cells that sense the two cues at the same time and average them with a unique sensing kernel $\gamma$. In the next section, we briefly present some preliminaries concerning asymptotic limit procedures and, then, we derive the hydrodynamics models in four different scenarios. We compare the macroscopic settings obtained from the two different transition probabilities described in this section.

## Derivation of the Hydrodynamic Models

### Preliminaries on Asymptotic Limit Procedures

In order to investigate the overall trend of the system, the macroscopic behavior is typically analyzed.

Precisely, we are interested in the evolution of $\rho (t,\mathbf{x})$ in the emerging regime of the system that may correspond to a diffusion-driven regime or to a drift-driven regime. Therefore, we shall consider a diffusive or a hydrodynamic scaling of the transport equation (4) with (5), respectively, resulting from a proper non-dimensionalization of the system. Diffusive and hydrodynamic limits for transport equations with velocity jump processes have been widely treated by Hillen and Othmer (2000); Othmer and Hillen (2002); Hillen (2006); Loy and Preziosi (2020); Bellomo et al. (2007); Filbet et al. (2005). For the reader’s convenience, in the present section we briefly report a well-known technique based on Hilbert expansions that are aimed at performing the diffusive and hyperbolic limits of our transport models that we shall present in the following sections. Formally, we introduce a small parameter $\epsilon \ll 1$ and we re-scale the spatial variable as

16 $$\begin{aligned} \varvec{\xi }=\epsilon \mathbf{x}, \end{aligned}$$

being $\varvec{\xi }$ the macroscopic spatial variable. According to the other characteristic quantities of the system and up to an appropriate nondimensionlization, the macroscopic time scale $\tau$ will be

17 $$\begin{aligned} \tau =\epsilon ^2 t, \end{aligned}$$

corresponding to the parabolic scaling in the case of a diffusion dominated phenomenon, or

18 $$\begin{aligned} \tau =\epsilon t, \end{aligned}$$

corresponding to the hyperbolic scaling in the case of a drift driven phenomenon. As widely treated by Othmer and Hillen (2002), we consider a framework in which, up to the spatial scaling (16), we can expand the transition probability as

$$\begin{aligned} T(\varvec{\xi },v,\hat{\mathbf{v}})=T_0(\varvec{\xi },v,\hat{\mathbf{v}})+\epsilon T_1(\varvec{\xi },v,\hat{\mathbf{v}})+{\mathcal {O}}(\epsilon ^2). \end{aligned}$$

This means that there are different orders of bias. If we assume that $\mu ={\mathcal {O}}(1)$, we denote by ${\mathcal {J}}^0$ and ${\mathcal {J}}^1$ the corresponding operators defined by $T_0$ and $T_1$, respectively, and we assume that

19a $$\begin{aligned} \displaystyle \int _{{\mathbb {S}}^{d-1}}\int _0^{V} T_0(\varvec{\xi }, v, \hat{\mathbf{v}}) \, dv d\hat{\mathbf{v}}=1, \end{aligned}$$

and

19b $$\begin{aligned} \displaystyle \int _{{\mathbb {S}}^{d-1}}\int _0^{V} T_i(\varvec{\xi }, v, \hat{\mathbf{v}}) \, dv d\hat{\mathbf{v}}=0 \quad \forall i \ge 1 . \end{aligned}$$

The corresponding first and second order moments are given by

20 $$\begin{aligned} \mathbf{U}_T^i(\varvec{\xi })=\int _{{\mathbb {S}}^{d-1}}\int _0^{V} T_i (\varvec{\xi },v,\hat{\mathbf{v}})\mathbf{v}\, dv d\hat{\mathbf{v}}\end{aligned}$$

and

21 $$\begin{aligned} {\mathbb {D}}_T^i(\varvec{\xi })=\int _{{\mathbb {S}}^{d-1}}\int _0^{V} T_i(\varvec{\xi }, v,\hat{\mathbf{v}}) (\mathbf{v}-\mathbf{U}^i_T)\otimes (\mathbf{v}-\mathbf{U}^i_T) dv \, d\hat{\mathbf{v}}\,. \end{aligned}$$

Considering the Hilbert expansion of the distribution function *p*

22 $$\begin{aligned} p=p_0+\epsilon p_1 +{\mathcal {O}}(\epsilon ^2)\,, \end{aligned}$$

if there is conservation of mass, we have that all the mass is in $p_0$ (Hillen and Othmer 2000), ${ i.e.}$,

23 $$\begin{aligned} \rho _0=\rho , \quad \rho _i=0 \quad \forall i \ge 1 \, , \end{aligned}$$

where ${\rho _i=\int _{{\mathbb {S}}^{d-1}}\int _0^{V} p_i \, dv\, d{\hat{\mathbf{v}}}}$. Furthermore, for performing the diffusive limit we shall assume that ${\int _{{\mathbb {S}}^{d-1}}\int _0^{V} p_i \,\mathbf{v}\, dv\, d{\hat{\mathbf{v}}} =0 } \quad \forall i \ge 2$ (Hillen and Othmer 2000). The fundamental property for performing the diffusive limit requires

24 $$\begin{aligned} \mathbf{U}^0_T=0, \end{aligned}$$

meaning that the leading order of the drift velocity vanishes. This is coherent with the fact that the time scale $\tau =\epsilon ^2 t$ is chosen because macroscopically the phenomenon is diffusion-driven.

Equation (4), rescaled according to (16)-(17), reads

25 $$\begin{aligned} \begin{aligned} \epsilon ^2\dfrac{\partial p}{\partial \tau }(\tau ,\mathbf{\varvec{\xi }},v,\hat{\mathbf{v}}) + \epsilon \mathbf{v}\cdot \nabla p(\tau ,\mathbf{\varvec{\xi }},v,\hat{\mathbf{v}})=&{\mathcal {J}}^0[p]+\epsilon {\mathcal {J}}^1[p] +{\mathcal {O}}(\epsilon ^2) \, , \end{aligned} \end{aligned}$$

where ${\mathcal {J}}^i[p]$ represents the turning operator defined by $T_i(\varvec{\xi }, v,\hat{\mathbf{v}})$. Equating the terms of equal order in $\epsilon$, we obtain the following system of equations.

In $$\epsilon ^{0}$$:

26 $$\begin{aligned} {\mathcal {J}}^0[p_{0}](\varvec{\xi },v,\hat{\mathbf{v}})\equiv \mu \Big ( \rho _0 T_0(\mathbf{\varvec{\xi }},v,\hat{\mathbf{v}})-p_{0}(\tau ,\mathbf{\varvec{\xi }},v,\hat{\mathbf{v}}) \Big )=0 \end{aligned}$$

In $$\epsilon ^{1}$$:

27 $$\begin{aligned} \begin{aligned} \nabla \cdot \big ( p_{0}(\tau , \mathbf{\varvec{\xi }},v,\hat{\mathbf{v}})\mathbf{v}\big )&={\mathcal {J}}^0[p_1](\tau ,\mathbf{\varvec{\xi }},v,\hat{\mathbf{v}}) +{\mathcal {J}}^1[p_0](\tau ,\mathbf{\varvec{\xi }},v,\hat{\mathbf{v}})\\&=\mu \big (\rho _1 T_0(\mathbf{\varvec{\xi }},v,\hat{\mathbf{v}}) -p_1(\tau ,\mathbf{\varvec{\xi }},v,\hat{\mathbf{v}})\big ) + \mu \rho _0 T_1(\mathbf{\varvec{\xi }},v,\hat{\mathbf{v}}) \end{aligned} \end{aligned}$$

In $$\epsilon ^{2}$$:

28 $$\begin{aligned} \dfrac{\partial }{\partial \tau }p_{0}(\mathbf{\varvec{\xi }},v,\hat{\mathbf{v}})+\nabla \cdot \big (p_{1}(\mathbf{\varvec{\xi }},v,\hat{\mathbf{v}})\mathbf{v}\big )= & {} {\mathcal {J}}^0[p_2](\mathbf{\varvec{\xi }},v,\hat{\mathbf{v}}) +{\mathcal {J}}^1[p_1](\mathbf{\varvec{\xi }},v,\hat{\mathbf{v}})\nonumber \\&+{\mathcal {J}}^2[p_0](\mathbf{\varvec{\xi }},v,\hat{\mathbf{v}}) \end{aligned}$$

Eq. (26) implies

29 $$\begin{aligned} p_0(\tau , \mathbf{\varvec{\xi }},v,\hat{\mathbf{v}})=\rho _0(\tau ,\mathbf{\varvec{\xi }})T_0(\mathbf{\varvec{\xi }},v,\hat{\mathbf{v}}) \end{aligned}$$

that is the equilibrium state of order zero. From Eq. (27), by inverting ${\mathcal {J}}^0$, we get

30 $$\begin{aligned} p_{1}(\mathbf{\varvec{\xi }},v,\hat{\mathbf{v}})=-\dfrac{1}{\mu } \nabla \cdot \big (\mathbf{v}p_{0}\big )+ \rho _0 T_1(\mathbf{\varvec{\xi }},v,\hat{\mathbf{v}}) \, . \end{aligned}$$

Precisely, the functional solvability condition necessary for inverting ${\mathcal {J}}^0$ is

31 $$\begin{aligned} \int _{{\mathbb {S}}^{d-1}}\int _0^{V}\left[ - \nabla \cdot \dfrac{1}{\mu } \big (\mathbf{v}p_{0} \big )+ \rho _0 T_1(\mathbf{\varvec{\xi }},v,\hat{\mathbf{v}})\right] \, dv d\hat{\mathbf{v}}=0 \!\!\!\!\qquad \textit{for}\,\,\,a.e.\,\,\, \varvec{\xi }, \end{aligned}$$

which is satisfied because (24) and (19b) are satisfied.

Integrating (28) over ${\mathbb {S}}^{d-1}\times [0,V]$, we obtain the macroscopic diffusive limit given by (dropping the dependencies)

32 $$\begin{aligned} \dfrac{\partial }{\partial {\tau }} \rho +\nabla \cdot \left( \mathbf{U}_T^1 \rho \right) =\nabla \cdot \left[ \dfrac{1}{\mu } \nabla \cdot \left( {\mathbb {D}}_T^0 \rho \right) \right] \,, \end{aligned}$$

being ${\mathbb {D}}_T^0(\varvec{\xi })$ the diffusion motility tensor. Equation (32) is a diffusion–advection equation, where $\mathbf{U}_T^1$ is the drift velocity of first order. If (24) does not hold, a hyperbolic scaling is more appropriate. In this case, rescaling the variables as in (16) and (18), the transport equation (4) reads

33 $$\begin{aligned} \begin{array}{lr} \epsilon \dfrac{\partial p}{\partial \tau }(\mathbf{\varvec{\xi }},v,\hat{\mathbf{v}}) \!+ \!\epsilon \mathbf{v}\cdot \nabla p(\mathbf{\varvec{\xi }},v,\hat{\mathbf{v}}) \!=\! \mu \, \left[ \rho \big (T_0(\mathbf{\varvec{\xi }},v,\hat{\mathbf{v}})\!+\!\epsilon T_1(\mathbf{\varvec{\xi }},v,\hat{\mathbf{v}})\big ) - p(\mathbf{\varvec{\xi }},v,\hat{\mathbf{v}}) \right] \, . \end{array} \end{aligned}$$

Since the equilibrium state is the same as before, we consider the Chapman–Enskog expansion of *p* in the form

34 $$\begin{aligned} p(\mathbf{\varvec{\xi }},v,\hat{\mathbf{v}})= \rho _{0} T_0(\mathbf{\varvec{\xi }},v,\hat{\mathbf{v}}) + \epsilon g +{\mathcal {O}}(\epsilon ^2). \end{aligned}$$

where $\int _{{\mathbb {S}}^{d-1}}\int _0^{V} g \, dv d\hat{\mathbf{v}}=0$. Substituting (34) in (33) and integrating the equation at the order $\epsilon ^{1}$ over ${\mathbb {S}}^{d-1}\times [0,V]$, we obtain

35 $$\begin{aligned} \dfrac{\partial }{\partial {\tau }}\rho +\nabla \cdot \left( \rho \mathbf{U}_T^0 \right) =0\,. \end{aligned}$$

This is an advection equation modeling a drift driven phenomenon. We address the reader to Loy and Preziosi (2020) for further details about the derivation of the macroscopic model. Concerning the boundary conditions (Plaza 2019), at the macroscopic level no flux boundary conditions for the diffusive limit read

$$\begin{aligned} \Big ( {\mathbb {D}}_T^0\nabla \rho -\rho \mathbf{U}_T^1\Big )\cdot \mathbf{n}=0, \quad \mathrm{on} \quad \partial \Omega , \end{aligned}$$

being **n** the outward normal to the boundary, whilst for the hyperbolic limit they are given by

$$\begin{aligned} \mathbf{U}_T^0\cdot \mathbf{n}=0, \quad \mathrm{on} \quad \partial \Omega . \end{aligned}$$

### Mesoscopic Analysis of Two Non-Local External Cues

In line with the analysis proposed by Loy and Preziosi (2020, 2020), in order to qualitatively evaluate the effects of the non-locality at the macroscopic level, we study the impact of both directional cues ${\mathcal {S}}$ and *q* with respect to the size of the cell (which is related to the cell sensing radius *R*). Precisely, the following analysis refers to spatially heterogeneous fiber networks and chemicals, as for homogeneous cues there is a loss of significance in considering a non-local sensing. As in Loy and Preziosi (2020), we introduce the characteristic length of variation of ${\mathcal {S}}$

36 $$\begin{aligned} l_{{\mathcal {S}}}:=\dfrac{1}{\max \limits _{\mathbf{x}\in \Omega } \frac{|\nabla {\mathcal {S}}|}{{\mathcal {S}}}}\,. \end{aligned}$$

It allows to approximate ${\mathcal {S}}(\mathbf{x}+\lambda \hat{\mathbf{v}})$ with a positive quantity

37 $$\begin{aligned} {\mathcal {S}}(\mathbf{x}+\lambda \hat{\mathbf{v}}) \sim {\mathcal {S}}(\mathbf{x})+\lambda \nabla {\mathcal {S}}\cdot \hat{\mathbf{v}}\ge 0 \quad \forall \lambda \le R \quad \textit{if} \quad R<l_{{\mathcal {S}}} \end{aligned}$$

where we neglect higher-order terms in $\lambda$. Beside the above defined characteristic length of variation of the chemoattractant, in the present work we define an analogue quantity for the fibers distribution. We define in particular

38 $$\begin{aligned} l_q:=\dfrac{1}{\max \limits _{\mathbf{x}\in \Omega }\, \max \limits _{\hat{\mathbf{v}}\in {\mathbb {S}}^{d-1}}\frac{|\nabla q \cdot \hat{\mathbf{v}}|}{q}}\,. \end{aligned}$$

In this case, we can approximate $q(\mathbf{x}+\lambda \hat{\mathbf{v}},\hat{\mathbf{v}})$ with a positive quantity

39 $$\begin{aligned} q(\mathbf{x}+\lambda \hat{\mathbf{v}}, \hat{\mathbf{v}}) \sim q(\mathbf{x},\hat{\mathbf{v}})+\lambda \nabla q \cdot \hat{\mathbf{v}}\ge 0 \quad \forall \lambda< R \quad \textit{if} \quad R<l_q\,. \end{aligned}$$

This definition of $l_q$ takes into account the variation in directionality of the fibers in space, which is what actually influences cell orientation, more than the spatial variation in the density of the ECM. We analyze the possible scenarios depending on the relation between *R*, $l_{\mathcal {S}}$ and $l_q$. For this purpose, let us introduce the parameters

40 $$\begin{aligned} \eta _{{\mathcal {S}}}:=\dfrac{R}{l_{{\mathcal {S}}}}\, \end{aligned}$$

and

41 $$\begin{aligned} \eta _q:=\dfrac{R}{l_q} \end{aligned}$$

that quantify the cell measuring capability with respect to the characteristic lengths of variation of the guidance cues ${\mathcal {S}}$ and *q*. In particular, $\eta _i<1, \,\, i=q, {\mathcal {S}}$, means that the sensing radius is smaller than the characteristic length of variation of *q* (${\mathcal {S}}$, respectively) and, thus, a single instantaneous sensing of the cell is not capable of catching the total spatial variability of *q* (${\mathcal {S}}$, respectively). On the other hand, if $\eta _i>1,\, \,i=q, {\mathcal {S}}$, the sensing radius is large enough to capture the spatial variability of *q* (${\mathcal {S}}$, respectively). If we consider the two cues separately, in the first case we expect that the sensing of *q* (${\mathcal {S}}$, respectively) induces a diffusive behavior, while in the second scenario the overall behavior induced by *q* (${\mathcal {S}}$, respectively) is drift driven. However, as we are considering the two guidance cues simultaneously affecting cell polarization, we have to take into account four limit cases:

*i*): *fast variation of both external cues*: $\eta _q, \eta _{{\mathcal {S}}} \gg 1$;
*ii*): *slow variation of both external cues*: $\eta _q, \eta _{{\mathcal {S}}} \ll 1$;
*iii*): *fast* *q* *and slow* ${\mathcal {S}}$ *variation*: $\eta _{{\mathcal {S}}} \ll 1, \eta _q\gg 1$;
*iv*): *fast* ${\mathcal {S}}$ *and slow* *q* *variation*: $\eta _{{\mathcal {S}}}\gg 1, \eta _q\ll 1$.

We remark that the concept of fast and slow variation refers to the cell sensing capability and it is intended with respect to the relation between cell sensing radius and characteristic variation length of the cues.

In the next section, we analyze the macroscopic trends in the cases $$i)-iv)$$. Precisely, in case (*i*), a Taylor expansion for both cues cannot be used, since there is no guarantee that the first order approximations are positive, as well as in case (*iii*) and (*iv*) for *q* and $${\mathcal {S}}$$, respectively. A priori, in order to quantify the relative contribution of chemotaxis to contact guidance, we introduce the parameter

42 $$\begin{aligned} \eta =\dfrac{\eta _q}{\eta _{{\mathcal {S}}}} \end{aligned}$$

that is larger than 1 if contact guidance prevails, whilst it is smaller then 1 if chemotaxis is stronger. Due to (41) and (40), we have that, despite its definition, $\eta$ does not depend on the size and sensing capability of the cell, but only on the characteristics of the cues, as $\eta =\eta _q/\eta _{{\mathcal {S}}}=l_{{\mathcal {S}}}/l_q$. In particular, if $l_{{\mathcal {S}}}$ is larger than $l_q$, ${ i.e.}$, $\eta >1$, it means that the gradient of *q* is steeper than the one of ${\mathcal {S}}$, thus enhancing a stronger effect of contact guidance on the dynamics. We may also observe that in the case of *fast* *q* *and slow* ${\mathcal {S}}$ *variation* (case (*iii*)) we always have $\eta >1$ while in the case of *fast* ${\mathcal {S}}$ *and slow* *q* *variation* (case (*iv*)) we always have $\eta <1$, ${ i.e.}$, contact guidance is weaker than chemotaxis.

### Amoeboid Motion and Chemotaxis: Non-Local Independent Sensing

We first consider the non-local independent sensing case (4)-(5) with (14). We recall the expression of the transition probability

$$\begin{aligned} T[q,{\mathcal {S}}](\mathbf{x}, v,\hat{\mathbf{v}})=c(\mathbf{x})\int _{{\mathbb {R}}_+}\gamma _{{\scriptscriptstyle {\mathcal {S}}}}(\lambda ) {\mathcal {S}}(\mathbf{x}+\lambda \hat{\mathbf{v}})\, d\lambda \, \int _{{\mathbb {R}}_+}\gamma _q(\lambda )\,q(\mathbf{x}+\lambda \hat{\mathbf{v}},\hat{\mathbf{v}})\,d\lambda \, \psi (v)\,. \end{aligned}$$

The average of *T*, which is the equilibrium velocity of the cell population, is given by

43 $$\begin{aligned} \mathbf{U}_T(\mathbf{x})=c(\mathbf{x})\,{\bar{U}} \int _{{\mathbb {S}}^{d-1}}\hat{\mathbf{v}}\left( \int _{{\mathbb {R}}_+}\gamma _{{\scriptscriptstyle {\mathcal {S}}}}(\lambda ) {\mathcal {S}}(\mathbf{x}+\lambda \hat{\mathbf{v}}) \,d\lambda \, \int _{{\mathbb {R}}_+}\gamma _q(\lambda )\,q(\mathbf{x}+\lambda \hat{\mathbf{v}},\hat{\mathbf{v}})\,d\lambda \right) d\hat{\mathbf{v}}\,.\nonumber \\ \end{aligned}$$

#### Case (*i*): *Fast Variation of Both External Cues*

In this case, we shall choose

$$\begin{aligned} \epsilon =\min \left\{ \frac{1}{\eta _q}, \frac{1}{\eta _{\mathcal {S}}} \right\} \,. \end{aligned}$$

As a consequence of the fact that *T* cannot be expanded in powers of $\epsilon$ after the re-scaling with (16), we have that the equilibrium velocity $\mathbf{U}_T$ is directly given by (43). Therefore, we perform a hyperbolic scaling that leads to the following macroscopic equation for the cell density:

44 $$\begin{aligned} \dfrac{\partial }{\partial \tau } \rho (\tau ,\varvec{\xi })+ \nabla \cdot (\rho (\tau ,\varvec{\xi }) \mathbf{U}_T(\varvec{\xi }))=0\,, \end{aligned}$$

with $\mathbf{U}_T(\varvec{\xi })$ given by the re-scaling of (43) with (16).

#### Case (*ii*): *Slow Variation of Both External Cues*

In this case, we can expand both ${\mathcal {S}}(\mathbf{x}+\lambda \hat{\mathbf{v}})$ and $q(\mathbf{x}+\lambda \hat{\mathbf{v}},\hat{\mathbf{v}})$ and consider the approximations (37) and (39) for $\lambda < \min \lbrace l_q,l_{\mathcal {S}}\rbrace$. Thus, we approximate the transition probability by substituting (37) and (39) in (14), and we obtain the following approximation for the turning kernel $T[q,{\mathcal {S}}]:$

45 $$\begin{aligned} \begin{aligned}&T[q,{\mathcal {S}}](\mathbf{x}, v,\hat{\mathbf{v}})\\&\quad =c(\mathbf{x})\Big [\Gamma _0^{\mathcal {S}}\,\Gamma _0^q\,{\mathcal {S}}(\mathbf{x})\,q(\mathbf{x},\hat{\mathbf{v}})+\Gamma _0^{\mathcal {S}}\,\Gamma _1^q\,{\mathcal {S}}(\mathbf{x})\,\nabla q \cdot \hat{\mathbf{v}}+\Gamma _1^{\mathcal {S}}\,\Gamma _0^q\,q(\mathbf{x},\hat{\mathbf{v}})\,\nabla {\mathcal {S}}\cdot \hat{\mathbf{v}}\Big ]\psi (v)\, \end{aligned}\nonumber \\ \end{aligned}$$

where we neglect higher orders terms in $\lambda$. In the latter,

$$\begin{aligned} c(\mathbf{x})=\dfrac{1}{{\mathcal {S}}(\mathbf{x})\,\Gamma _0^{\mathcal {S}}\,\Gamma _0^q} \end{aligned}$$

and

$$\begin{aligned} \Gamma _i^{\mathcal {S}}:=\int _{{\mathbb {R}}_+}\lambda ^i\gamma _{{\scriptscriptstyle {\mathcal {S}}}}(\lambda )\,d\lambda \quad \quad i=0,1 \\ \Gamma _i^q:=\int _{{\mathbb {R}}_+}\lambda ^i\gamma _q(\lambda )\,d\lambda \quad \quad i=0,1\,. \end{aligned}$$

The quantities $\Gamma _0^q, \Gamma _0^{\mathcal {S}}$ are the weighted (by $\gamma _q, \gamma _{{\scriptscriptstyle {\mathcal {S}}}}$) measures of the sensed linear tracts in every direction, whilst $\Gamma _1^q, \Gamma _1^{\mathcal {S}}$ are the averages of $\gamma _q, \gamma _{{\scriptscriptstyle {\mathcal {S}}}}$ on [0, *R*]. Then, we introduce the small parameter

46 $$\begin{aligned} \epsilon =\min \lbrace \eta _q, \eta _{\mathcal {S}}\rbrace \end{aligned}$$

and re-scale the space variable as $\varvec{\xi }=\epsilon \mathbf{x}$, getting

47 $$\begin{aligned} T_0[q,{\mathcal {S}}](\varvec{\xi },v,\hat{\mathbf{v}})= q(\varvec{\xi },\hat{\mathbf{v}}) \psi (v)\,. \end{aligned}$$

This expression means that the equilibrium is determined by the fiber distribution. Moreover,

$$\begin{aligned} T_1[q,{\mathcal {S}}](\varvec{\xi },v,\hat{\mathbf{v}})=\left[ \Gamma ^q\,\nabla q \cdot \hat{\mathbf{v}}+\Gamma ^{\mathcal {S}}\,q(\varvec{\xi },\hat{\mathbf{v}})\,\dfrac{\nabla {\mathcal {S}}}{{\mathcal {S}}(\varvec{\xi })}\cdot \hat{\mathbf{v}}\right] \psi (v) \end{aligned}$$

where

$$\begin{aligned} \Gamma ^{\mathcal {S}}:=\dfrac{\Gamma _1^{\mathcal {S}}}{\Gamma _0^{\mathcal {S}}}\text {,}\qquad \Gamma ^q:=\dfrac{\Gamma _1^q}{\Gamma _0^q}\,. \end{aligned}$$

It can be easily checked that $T_0$ and $T_1$ verify (19a) and (19b), respectively. Because of (12) and (47), we get $\mathbf{U}_0^T(\varvec{\xi })=0$, meaning that we are in a diffusive regime. The diffusive limit leads to the advection-diffusion equation (32). The explicit form for the zeroth-order macroscopic diffusion tensor is

48 $$\begin{aligned} {\mathbb {D}}_T^0(\varvec{\xi })=D\int _{{\mathbb {S}}^{d-1}}q(\varvec{\xi },\hat{\mathbf{v}})\hat{\mathbf{v}}\otimes \hat{\mathbf{v}}\,d\hat{\mathbf{v}}=D\,{\mathbb {D}}_q(\varvec{\xi })\,, \end{aligned}$$

while for the macroscopic first-order velocity is

49 $$\begin{aligned} \begin{aligned} \mathbf{U}_T^1(\varvec{\xi })&={\bar{U}}\int _{{\mathbb {S}}^{d-1}}\left( \Gamma ^q\,\nabla q\cdot \hat{\mathbf{v}}+ \Gamma ^{\mathcal {S}}\,\dfrac{\nabla {\mathcal {S}}}{{\mathcal {S}}(\varvec{\xi })}\cdot \hat{\mathbf{v}}\,q(\varvec{\xi },\hat{\mathbf{v}})\right) \hat{\mathbf{v}}d\hat{\mathbf{v}}\\&={\bar{U}}\,\Gamma ^q\int _{{\mathbb {S}}^{d-1}}\left( \nabla q\cdot \hat{\mathbf{v}}\right) \, \hat{\mathbf{v}}d\hat{\mathbf{v}}+{\bar{U}}\,\Gamma ^{\mathcal {S}}\,\dfrac{\nabla {\mathcal {S}}}{{\mathcal {S}}} \int _{{\mathbb {S}}^{d-1}}\hat{\mathbf{v}}\otimes \hat{\mathbf{v}}\,q(\varvec{\xi },\hat{\mathbf{v}}) d\hat{\mathbf{v}}\\&={\bar{U}}\,\left[ \Gamma ^q\,\nabla \cdot {\mathbb {D}}_q+\,\Gamma ^{\mathcal {S}}\,{\mathbb {D}}_q\,\dfrac{\nabla {\mathcal {S}}}{{\mathcal {S}}}\right] . \end{aligned} \end{aligned}$$

Therefore, the resulting diffusion–advection equation reads (dropping the dependencies)

50 $$\begin{aligned} \dfrac{\partial }{\partial \tau }\rho + \nabla \cdot \left[ \left( \chi ^{\mathcal {S}}\,{\mathbb {D}}_q\nabla {\mathcal {S}}+\chi ^q\nabla \cdot {\mathbb {D}}_q\right) \rho \right] =\nabla \cdot \left[ \dfrac{1}{\mu }\,\nabla \cdot \big (D\,{\mathbb {D}}_q\,\rho \big )\right] \,, \end{aligned}$$

where

51 $$\begin{aligned} \chi ^{\mathcal {S}}(\varvec{\xi }):=\dfrac{{\bar{U}}\,\Gamma ^{\mathcal {S}}\,}{{\mathcal {S}}(\varvec{\xi })}\quad \text {,}\quad \chi ^q:={\bar{U}}\,\Gamma ^q \end{aligned}$$

are the sensitivities. The diffusion represented by the cell motility tensor (48) only depends on the fibers distribution, while the advective term has two contributions differently weighted by the sensitivities in (51). We remark that, in the diffusive regime we obtain the same macroscopic behavior postulated by Keller and Segel (1970), with the logarithmic chemotactic sensitivity $\chi _{{\mathcal {S}}}$ given in (51). The term ${\mathbb {D}}_q\nabla {\mathcal {S}}$ depends on both the fibers distribution and the chemotactic field; it never vanishes if $\nabla {\mathcal {S}}$ is not the null vector, since it may be proved that ${\mathbb {D}}_q$ is invertible. In the isotropic case, corresponding to randomly disposed fibers, ${ i.e.}$, when ${\mathbb {D}}_q$ is proportional to the identity matrix, then ${\mathbb {D}}_q\nabla {\mathcal {S}}$ is parallel to $\nabla {\mathcal {S}}$, which, thus, represents the anisotropy direction. On the other hand, when ${\mathbb {D}}_q$ is anisotropic and $\nabla {\mathcal {S}}$ is not parallel to the leading eigenvector of ${\mathbb {D}}_q$, then the migration does not follow the dominant direction of the fibers, but rather its projection on $\nabla {\mathcal {S}}$. The second contribution in the drift term, ${ i.e.}$, $\nabla \cdot {\mathbb {D}}_q$, is a measure of the velocity field induced by the spatial variation of the fiber distribution, which determines the cell microscopic velocities. This term vanishes if the fibers distribution is homogeneous in space. However, if *q* is homogeneous in space, even in case of competing cues, ${ i.e.}$, $\mathbf{E }_{q}\perp \nabla {\mathcal {S}}$, in general the advective term $\mathbf{U}_T^1$ does not vanish. Instead, in case of cooperating cues, ${ i.e.}$, when $\nabla {\mathcal {S}}$ is an eigenvector of ${\mathbb {D}}_q$ with eigenvalue $D_{\nabla {\mathcal {S}}}$, migration is in direction $\nabla {\mathcal {S}}$ with a kinetic factor $\chi _{{\scriptscriptstyle {\mathcal {S}}}} D_{\nabla {\mathcal {S}}}$. In intermediate scenarios, migration happens in the projection ${\mathbb {D}}_q\nabla {\mathcal {S}}$, but, if *q* is not homogeneous, the dynamics is more complex and, even in case of cooperation, we cannot conclude anything about additivity effects.

#### Case (*iii*): *Fastqand Slow*${\mathcal {S}}$*Variation*

In this case, we can only expand with Taylor series the chemoattractant, as in (37), and the turning kernel (14) can be approximated as

52 $$\begin{aligned} T[q,{\mathcal {S}}](\mathbf{x}, v,\hat{\mathbf{v}})\displaystyle= & {} \displaystyle \!\left[ \dfrac{\Gamma _0^{\mathcal {S}}}{c_0(\mathbf{x})}\,{\mathcal {S}}(\mathbf{x})\displaystyle \!\!\int _{{\mathbb {R}}_+}\displaystyle \!\!\!\gamma _q(\lambda )q(\mathbf{x}+\lambda \hat{\mathbf{v}},\hat{\mathbf{v}})\,d\lambda +\dfrac{\Gamma _1^{\mathcal {S}}}{c_1(\mathbf{x})}\,(\nabla {\mathcal {S}}\cdot \hat{\mathbf{v}})\right. \nonumber \\&\left. \displaystyle \int _{{\mathbb {R}}_+}\displaystyle \!\!\!\gamma _q(\lambda )q(\mathbf{x}+\lambda \hat{\mathbf{v}},\hat{\mathbf{v}})\,d\lambda \right] \psi (v) \end{aligned}$$

where

$$\begin{aligned} c_0(\mathbf{x}):=2\int _{{\mathbb {S}}^{d-1}}\Gamma _0^{\mathcal {S}}\,S(\mathbf{x})\int _{{\mathbb {R}}_+}\gamma _{q}(\lambda )q(\mathbf{x}+\lambda \hat{\mathbf{v}},\hat{\mathbf{v}})\,d\lambda \,d\hat{\mathbf{v}}\end{aligned}$$

and

$$\begin{aligned} c_1(\mathbf{x}):=2\int _{{\mathbb {S}}^{d-1}}\Gamma _1^{\mathcal {S}}\,(\nabla S \cdot \hat{\mathbf{v}})\,\int _{{\mathbb {R}}_+}\gamma _{q}(\lambda )q(\mathbf{x}+\lambda \hat{\mathbf{v}},\hat{\mathbf{v}})\,d\lambda \,d\hat{\mathbf{v}}\,, \end{aligned}$$

are both different from zero. In this case we may choose

$$\begin{aligned} \epsilon =\min \left\{ \dfrac{1}{\eta _q}, \eta _{{\mathcal {S}}}\right\} , \end{aligned}$$

and, re-scaling the space variable as (16), we get $T[q,{\mathcal {S}}]=T_0[q,{\mathcal {S}}]$. Let us now introduce

53 $$\begin{aligned} {\tilde{q}}(\varvec{\xi },\hat{\mathbf{v}}):=\dfrac{1}{\Gamma _0^q}\int _{{\mathbb {R}}_+}\gamma _q(\lambda )q(\varvec{\xi }+\lambda \hat{\mathbf{v}},\hat{\mathbf{v}})\,d\lambda \end{aligned}$$

a new probability density distribution describing a non-local average of the fibers distribution according to the sensing kernel $\gamma _q$ and normalized by the measure of the sensed linear tract $\Gamma _0^q$ over the direction $\hat{\mathbf{v}}$. With this notation, we can define

54 $$\begin{aligned} {\mathbb {D}}_{{\tilde{q}}}^0(\varvec{\xi }):=\int _{{\mathbb {S}}^{d-1}}{\tilde{q}}(\varvec{\xi },\hat{\mathbf{v}})\,\hat{\mathbf{v}}\otimes \hat{\mathbf{v}}\,d\hat{\mathbf{v}}\,, \end{aligned}$$

which is the corresponding diffusion tensor of the fibers, and

55 $$\begin{aligned} {\mathbf {E}}_{{\tilde{q}}}(\varvec{\xi }):=\int _{{\mathbb {S}}^{d-1}}{\tilde{q}}(\varvec{\xi },\hat{\mathbf{v}})\, \hat{\mathbf{v}}\,d\hat{\mathbf{v}}\,, \end{aligned}$$

which is their corresponding mean direction. Precisely, both are defined taking into account the whole neighborhood sensed by the cells. In this case, $\mathbf{U}_T^0(\varvec{\xi })$ does not vanish in general in $\Omega$, as it is given by

56 $$\begin{aligned} \begin{aligned} \mathbf{U}_T^0(\varvec{\xi })&=\dfrac{{\bar{U}}\,\Gamma _0^{\mathcal {S}}}{c_0(\varvec{\xi })}\,S(\varvec{\xi })\,\int _{{\mathbb {R}}_+}\gamma _q(\lambda )\,\int _{{\mathbb {S}}^{d-1}}\hat{\mathbf{v}}\,q(\varvec{\xi }+\lambda \hat{\mathbf{v}},\hat{\mathbf{v}})\,d\hat{\mathbf{v}}\,d\lambda \\&\quad +\dfrac{{\bar{U}}\,\Gamma _1^{\mathcal {S}}}{c_1(\varvec{\xi })}\,\nabla {\mathcal {S}}\, \int _{{\mathbb {R}}_+}\gamma _q(\lambda )\,\int _{{\mathbb {S}}^{d-1}}\hat{\mathbf{v}}\otimes \hat{\mathbf{v}}\,q(\varvec{\xi }+\lambda \hat{\mathbf{v}},\hat{\mathbf{v}}) \,d\hat{\mathbf{v}}\,d\lambda ,\\&=\dfrac{{\bar{U}}\,\Gamma _0^{\mathcal {S}}\,\Gamma _0^q}{c_0(\varvec{\xi })}\,{\mathcal {S}}(\varvec{\xi })\,\,{\mathbf {E}}_{{\tilde{q}}}+\dfrac{{\bar{U}}\,\Gamma _1^{\mathcal {S}}\,\Gamma _0^q}{c_1(\varvec{\xi })}\,\nabla {\mathcal {S}}\,\,{\mathbb {D}}_{{\tilde{q}}}^0\,. \end{aligned} \end{aligned}$$

Therefore, we perform a hyperbolic limit that leads to the drift equation (35) with mean velocity (56). Precisely, this mean velocity is related to a non-local average of the diffusion tensor of the fibers ${\mathbb {D}}_{{\tilde{q}}}^0$ projected on $\nabla {\mathcal {S}}$, and a non-local average of the mean fiber direction depending on the local chemoattractant ${\mathcal {S}}$. In this case, there is not an evident additivity effect of the two cues and several scenarios are possible.

**Remark** In the particular case in which also the new introduced probability density ${\tilde{q}}$ has the same symmetry property Q3 (described in Sect. 2.2), or if we consider $\gamma _q=\delta _0(\lambda )$ (local sensing of the fibers), a parabolic scaling could be performed instead of a hyperbolic one. This would lead to a macroscopic diffusion–advection equation with mean velocity $U_T^1(\varvec{\xi })\propto {\mathbb {D}}^0_q\nabla {\mathcal {S}}$. Without chemotaxis, we would recover the classical model for contact guidance (Hillen 2006), which gives rise to a fully anisotropic diffusive equation at the macroscopic level. The presence of a non-local chemoattractant, even when $R<l_{\mathcal {S}}$, is responsible for the emergence of the drift correction term.

#### Case (*iv*): *Fast*${\mathcal {S}}$*and SlowqVariation*

This last case only allows for the Taylor expansion of the distribution function *q*, as in (39). Therefore, the turning kernel can be approximated as

57 $$\begin{aligned} \begin{aligned} T[q,{\mathcal {S}}](\mathbf{x}, v,\hat{\mathbf{v}})&=\left[ \dfrac{\Gamma _0^q}{c_0(\mathbf{x})}\,q(\mathbf{x},\hat{\mathbf{v}})\,\displaystyle \!\!\!\int _{{\mathbb {R}}_+}\displaystyle \!\!\!\!\gamma _{{\scriptscriptstyle {\mathcal {S}}}}(\lambda ){\mathcal {S}}(\mathbf{x}+\lambda \hat{\mathbf{v}})\,d\lambda +\dfrac{\Gamma _1^q}{c_1(\mathbf{x})}\,(\nabla q\cdot \hat{\mathbf{v}})\right. \\&\qquad \quad \left. \displaystyle \!\!\times \int _{{\mathbb {R}}_+}\displaystyle \!\!\!\!\gamma _{{\scriptscriptstyle {\mathcal {S}}}}(\lambda ){\mathcal {S}}(\mathbf{x}+\lambda \hat{\mathbf{v}})\,d\lambda \right] \psi (v) \end{aligned} \end{aligned}$$

where

$$\begin{aligned} c_0(\mathbf{x}):=2\int _{{\mathbb {S}}^{d-1}}\Gamma _0^q\,q(\mathbf{x},\hat{\mathbf{v}})\int _{{\mathbb {R}}_+}\gamma _{{\scriptscriptstyle {\mathcal {S}}}}(\lambda ){\mathcal {S}}(\mathbf{x}+\lambda \hat{\mathbf{v}})\,d\lambda \,d\hat{\mathbf{v}}\end{aligned}$$

and

$$\begin{aligned} c_1(\mathbf{x}):=2\int _{{\mathbb {S}}^{d-1}}\Gamma _1^q\,(\nabla q \cdot \hat{\mathbf{v}})\,\int _{{\mathbb {R}}_+}\gamma _{{\scriptscriptstyle {\mathcal {S}}}}(\lambda ){\mathcal {S}}(\mathbf{x}+\lambda \hat{\mathbf{v}})\,d\lambda \,d\hat{\mathbf{v}}\,, \end{aligned}$$

both different from zero. In this case we may choose

$$\begin{aligned} \epsilon =\min \left\{ \dfrac{1}{\eta _{{\mathcal {S}}}}, \eta _{q}\right\} \end{aligned}$$

and, by re-scaling (57) with (16), we get $T[q,{\mathcal {S}}]=T_0[q,{\mathcal {S}}]$. Hence $\mathbf{U}_T^0(\varvec{\xi })$ does not vanish in $\Omega$, as it is given by

58 $$\begin{aligned} \begin{aligned} \mathbf{U}_T^0(\varvec{\xi })&=\dfrac{{\bar{U}}\,\Gamma _0^q}{c_0(\varvec{\xi })}\,\int _{{\mathbb {S}}^{d-1}}\hat{\mathbf{v}}\,q(\varvec{\xi },\hat{\mathbf{v}})\,\,\int _{{\mathbb {R}}_+}\gamma _{{\scriptscriptstyle {\mathcal {S}}}}(\lambda )\,{\mathcal {S}}(\varvec{\xi }+\lambda \hat{\mathbf{v}})\,d\lambda \,d\hat{\mathbf{v}}\\&\quad \, +\dfrac{{\bar{U}}\,\Gamma _1^q}{c_1(\varvec{\xi })}\int _{{\mathbb {S}}^{d-1}}\hat{\mathbf{v}}\otimes \hat{\mathbf{v}}\,\nabla q \int _{{\mathbb {R}}_+} \gamma _{{\scriptscriptstyle {\mathcal {S}}}}(\lambda )\,{\mathcal {S}}(\varvec{\xi }+\lambda \hat{\mathbf{v}}) \, d\lambda \, d\hat{\mathbf{v}}\,, \end{aligned} \end{aligned}$$

and the macroscopic equation is given by (35). The mean velocity (58) is a linear combination of a non-local measure of the chemoattractant ${\mathcal {S}}$ over the fiber network and a non-local measure of ${\mathcal {S}}$ weighted by the directional average of the spatial variability of the fiber direction.

##### Remark

If we consider a local sensing for the chemoattractant, ${ i.e.}$, $\gamma _{{\scriptscriptstyle {\mathcal {S}}}}=\delta _0(\lambda )$, we obtain a macroscopic advection-diffusion equation. Here the macroscopic velocity would be induced by the spatial variation of the fiber direction distribution $\nabla \cdot {\mathbb {D}}_q$, and the measure of ${\mathcal {S}}$ does not affect the choice of the direction. In this case, if $\nabla q$ vanishes, the model would reduce to a fully anisotropic diffusive equation (Hillen 2006).

### Amoeboid Motion and Chemotaxis: Non-Local Dependent Sensing

Concerning the non-local dependent sensing case (4)–(5) with (15), we recall the expression of the transition probability

$$\begin{aligned} T[q,{\mathcal {S}}](\mathbf{x}, v,\hat{\mathbf{v}})=c(\mathbf{x})\int _{{\mathbb {R}}_+}\gamma (\lambda ) {\mathcal {S}}(\mathbf{x}+\lambda \hat{\mathbf{v}})\,q(\mathbf{x}+\lambda \hat{\mathbf{v}},\hat{\mathbf{v}})d\lambda \, \psi (v)\,, \end{aligned}$$

with

$$\begin{aligned} c(\mathbf{x}):=\int _{{\mathbb {S}}^{d-1}}\int _{{\mathbb {R}}_+}\gamma (\lambda ) {\mathcal {S}}(\mathbf{x}+\lambda \hat{\mathbf{v}})\,q(\mathbf{x}+\lambda \hat{\mathbf{v}},\hat{\mathbf{v}})d\lambda \,. \end{aligned}$$

The macroscopic velocity is here given by

59 $$\begin{aligned} \mathbf{U}_T(\mathbf{x})=c(\mathbf{x})\,{\bar{U}}\,\int _{{\mathbb {S}}^{d-1}}\hat{\mathbf{v}}\,\int _{{\mathbb {R}}_+}\gamma (\lambda ) {\mathcal {S}}(\mathbf{x}+\lambda \hat{\mathbf{v}}) \,q(\mathbf{x}+\lambda \hat{\mathbf{v}},\hat{\mathbf{v}})d\lambda \,d\hat{\mathbf{v}}\,. \end{aligned}$$

The macroscopic limits can be performed analogously to the previous section and using the techniques described in Sect. 3.1. The choice of the parameter $\epsilon$ for the cases $(i)-(iv)$ will be the same of the non-local independent sensing model, since it does not depend on the kind of model (independent or dependent sensing), but only on $\eta _{{\mathcal {S}}}$ and $\eta _{q}$, i.e., on the rapidity of variation of ${\mathcal {S}}$ and *q* with respect to the cell sensing capability.

#### Case (*i*): *Fast Variation of Both External Cues*

In this case, we cannot consider the expansions (39) and (37), and, thus, we cannot expand the turning kernel. Its non-vanishing average is given by (59). Therefore, we perform a hyperbolic limit leading to (35) with macroscopic velocity (59).

#### Case (*ii*): *Slow Variation of Both External Cues*

When, instead, the maximum sensing radius *R* is smaller than both characteristic variation lengths, we consider the positive expansions (39) and (37) and substitute them into (15). Neglecting the higher order terms in $\lambda$, we get the approximation

60 $$\begin{aligned} T[q,{\mathcal {S}}](\mathbf{x}, v,\hat{\mathbf{v}})=c(\mathbf{x})\Big [{\mathcal {S}}(\mathbf{x})\,\Gamma _0\,q(\mathbf{x},\hat{\mathbf{v}})+{\mathcal {S}}(\mathbf{x})\,\Gamma _1\,\nabla q \cdot \hat{\mathbf{v}}+\Gamma _1\,q(\mathbf{x},\hat{\mathbf{v}})\,\nabla {\mathcal {S}}\cdot \hat{\mathbf{v}}\Big ]\,\psi (v)\nonumber \\ \end{aligned}$$

with

$$\begin{aligned} c(\mathbf{x})=\dfrac{1}{{\mathcal {S}}(\mathbf{x})\,\Gamma _0} \end{aligned}$$

and

$$\begin{aligned} \Gamma _i:=\int _0^R\lambda ^i\gamma (\lambda )\,d\lambda \,,\qquad i=0,1\,. \end{aligned}$$

Re-scaling the space variable as in (16), we find

$$\begin{aligned} T_0[q,{\mathcal {S}}](\varvec{\xi },v,\hat{\mathbf{v}})=q(\varvec{\xi },\hat{\mathbf{v}})\psi (v) \end{aligned}$$

and

$$\begin{aligned} T_1[q,{\mathcal {S}}](\varvec{\xi },v,\hat{\mathbf{v}})=\Gamma \Big [\nabla q \cdot \hat{\mathbf{v}}+q(\varvec{\xi },\hat{\mathbf{v}})\,\dfrac{\nabla {\mathcal {S}}}{{\mathcal {S}}}\cdot \hat{\mathbf{v}}\Big ]\,\psi (v)\, \end{aligned}$$

with

$$\begin{aligned} \Gamma :=\dfrac{\Gamma _1}{\Gamma _0}. \end{aligned}$$

It can be easily checked that $T_0$ and $T_1$ verify (19a) and (19b), respectively. Therefore, $\mathbf{U}_0^T(\varvec{\xi })=0$, because of (12), and we can perform a diffusive scaling that leads to the zero-order macroscopic diffusion tensor

61 $$\begin{aligned} {\mathbb {D}}_T^0(\varvec{\xi })=D\,{\mathbb {D}}_q(\varvec{\xi })\,, \end{aligned}$$

and to the macroscopic first-order velocity

62 $$\begin{aligned} \mathbf{U}_T^1(\varvec{\xi })={\bar{U}}\,\Gamma \,\nabla \cdot {\mathbb {D}}_q(\varvec{\xi })+{\bar{U}}\,\Gamma \,{\mathbb {D}}_q(\varvec{\xi })\,\,\dfrac{\nabla {\mathcal {S}}}{{\mathcal {S}}}\,. \end{aligned}$$

The macroscopic advection-diffusion equation (32) now reads (dropping the dependencies)

63 $$\begin{aligned} \dfrac{\partial }{\partial \tau }\rho +\nabla \cdot \left[ \chi \,\left( \nabla \cdot {\mathbb {D}}_q\,+{\mathbb {D}}_q\dfrac{\nabla {\mathcal {S}}}{{\mathcal {S}}}\right) \,\rho \right] =\nabla \cdot \left[ \dfrac{1}{\mu }\,\nabla \cdot \big (D\,{\mathbb {D}}_q\,\rho \big )\right] \end{aligned}$$

where

$$\begin{aligned} \chi :={\bar{U}}\Gamma \,. \end{aligned}$$

Similar observations to the case (*ii*) of the non-local independent sensing model may be done, except that, here, there is a unique sensitivity $\chi$ that weights equally the two contributions in the advection term (62).

#### Case (*iii*): *Fastqand Slow*${\mathcal {S}}$*Variation*

In this case, we expand only the chemoattractant ${\mathcal {S}}(\mathbf{x}+\lambda \hat{\mathbf{v}})$, as in (37), and the turning kernel (15) can be approximated as

64 $$\begin{aligned} T[q,{\mathcal {S}}](\mathbf{x}, v,\hat{\mathbf{v}})= & {} \left[ \dfrac{1}{c_0(\mathbf{x})}\,{\mathcal {S}}(\mathbf{x})\int _{{\mathbb {R}}_+}\!\!\gamma (\lambda )q(\mathbf{x}+\lambda \hat{\mathbf{v}},\hat{\mathbf{v}})d\lambda +\dfrac{1}{c_1(\mathbf{x})}(\nabla {\mathcal {S}}\cdot \hat{\mathbf{v}})\right. \nonumber \\&\left. \int _{{\mathbb {R}}_+}\!\!\lambda \,\gamma (\lambda )q(\mathbf{x}+\lambda \hat{\mathbf{v}},\hat{\mathbf{v}})d\lambda \right] \psi (v) \end{aligned}$$

with

$$\begin{aligned} c_0(\mathbf{x}):=2\int _{{\mathbb {S}}^{d-1}}{\mathcal {S}}(\mathbf{x})\int _{{\mathbb {R}}_+}\gamma (\lambda )q(\mathbf{x}+\lambda \hat{\mathbf{v}},\hat{\mathbf{v}})\,d\lambda \,d\hat{\mathbf{v}}\end{aligned}$$

and

$$\begin{aligned} c_1(\mathbf{x}):=2\int _{{\mathbb {S}}^{d-1}}(\nabla {\mathcal {S}}\cdot \hat{\mathbf{v}})\,\int _{{\mathbb {R}}_+}\lambda \gamma (\lambda )q(\mathbf{x}+\lambda \hat{\mathbf{v}},\hat{\mathbf{v}})\,d\lambda \,d\hat{\mathbf{v}}\,, \end{aligned}$$

both different from zero. Re-scaling the space variable as (16), we find $T[q,{\mathcal {S}}]=T_0[q,{\mathcal {S}}]$. Let us define

65 $$\begin{aligned} \bar{{\mathbf {E}}}_{q}(\varvec{\xi })=\dfrac{1}{\Gamma _0}\int _{{\mathbb {S}}^{d-1}}\int _{{\mathbb {R}}_+}\gamma (\lambda )\,q(\varvec{\xi }+\lambda \hat{\mathbf{v}},\hat{\mathbf{v}})\,d\lambda \,\hat{\mathbf{v}}\,d\hat{\mathbf{v}}\,. \end{aligned}$$

and

66 $$\begin{aligned} \bar{{\mathbb {D}}}_q^1(\varvec{\xi })=\dfrac{1}{\Gamma _1}\int _{{\mathbb {S}}^{d-1}}\int _{{\mathbb {R}}_+}\lambda \gamma (\lambda )q(\varvec{\xi }+\lambda \hat{\mathbf{v}},\hat{\mathbf{v}})\,\hat{\mathbf{v}}\otimes \hat{\mathbf{v}}\,d\hat{\mathbf{v}}\,, \end{aligned}$$

mean fiber direction and diffusion tensor, respectively. They are related to the average, through the function $\gamma$, of the non-local fiber distribution evaluated on the whole neighborhood sensed by the cells. Hence, in this case, $\mathbf{U}_T^0(\varvec{\xi })$ does not vanish in general in $\Omega$, as it is given by

67 $$\begin{aligned} \begin{aligned} \mathbf{U}_T^0(\varvec{\xi })&=\dfrac{{\bar{U}}}{c_0(\varvec{\xi })}\,{\mathcal {S}}(\varvec{\xi })\,\int _{{\mathbb {R}}_+}\gamma (\lambda )\,\int _{{\mathbb {S}}^{d-1}}\hat{\mathbf{v}}q(\varvec{\xi }\,+\lambda \hat{\mathbf{v}},\hat{\mathbf{v}})\,d\hat{\mathbf{v}}\,d\lambda \\&+\dfrac{{\bar{U}}}{c_1(\varvec{\xi })}\,\nabla {\mathcal {S}}\, \int _{{\mathbb {R}}_+}\lambda \, \gamma (\lambda )\,\int _{{\mathbb {S}}^{d-1}}q(\varvec{\xi }+\lambda \hat{\mathbf{v}},\hat{\mathbf{v}})\hat{\mathbf{v}}\otimes \hat{\mathbf{v}}\,d\hat{\mathbf{v}}\,d\lambda ,\\&=\dfrac{{\bar{U}}\,\Gamma _0}{c_0(\varvec{\xi })}\,{\mathcal {S}}(\varvec{\xi })\,\bar{{\mathbf {E}}}_q(\varvec{\xi })+\dfrac{{\bar{U}}\,\Gamma _1}{c_1(\varvec{\xi })}\,\nabla {\mathcal {S}}\,\bar{{\mathbb {D}}}_q^1(\varvec{\xi })\,. \end{aligned} \end{aligned}$$

Therefore, the macroscopic advection equation has an expression analogous to (35) with macroscopic velocity (67), which represents a linear combination of a non-local weighted average of the diffusion tensor of the fibers $\bar{{\mathbb {D}}}_q^1$ projected on $\nabla {\mathcal {S}}$, and a non-local average of the mean fiber direction depending on the local chemoattractant ${\mathcal {S}}$. As for the independent sensing case, there is not a simple additivity effect of the two cues and several scenarios are possible.

#### Case (*iv*): *Fast*${\mathcal {S}}$*and SlowqVariation*

In this case, again, we can only consider the positive approximation (39), and the transition probability rewrites as

68 $$\begin{aligned} \begin{aligned} T[q,{\mathcal {S}}](\mathbf{x}, v,\hat{\mathbf{v}})=&\left[ \dfrac{1}{c_0(\mathbf{x})}q(\mathbf{x},\hat{\mathbf{v}})\,\int _{{\mathbb {R}}_+} \gamma (\lambda )\,{\mathcal {S}}(\mathbf{x}+\lambda \hat{\mathbf{v}})\,d\lambda \right. \\&\left. +\dfrac{1}{c_1(\mathbf{x})}\nabla q\cdot \hat{\mathbf{v}}\,\int _{{\mathbb {R}}_+}\lambda \,\gamma (\lambda )\,{\mathcal {S}}(\mathbf{x}+\lambda \hat{\mathbf{v}})\,d\lambda \right] \psi (v) \end{aligned} \end{aligned}$$

where

$$\begin{aligned} c_0(\mathbf{x}):=2\int _{{\mathbb {S}}^{d-1}}q(\mathbf{x},\hat{\mathbf{v}})\int _{{\mathbb {R}}_+}\gamma (\lambda ){\mathcal {S}}(\mathbf{x}+\lambda \hat{\mathbf{v}})\,d\lambda \,d\hat{\mathbf{v}}\end{aligned}$$

and

$$\begin{aligned} c_1(\mathbf{x}):= 2\int _{{\mathbb {S}}^{d-1}}(\nabla q \cdot \hat{\mathbf{v}})\,\int _{{\mathbb {R}}_+}\lambda \,\gamma (\lambda ){\mathcal {S}}(\mathbf{x}+\lambda \hat{\mathbf{v}})\,d\lambda \,d\hat{\mathbf{v}}\,, \end{aligned}$$

both different from zero. As before, by re-scaling (68) with (16), we get $T[q,{\mathcal {S}}]=T_0[q,{\mathcal {S}}]$ and we have that the average velocity $\mathbf{U}_T^0=\mathbf{U}_T\ne 0$. In particular, it is given by

69 $$\begin{aligned} \begin{aligned} \mathbf{U}_T^0(\varvec{\xi }):&=\dfrac{{\bar{U}}}{c_0(\varvec{\xi })}\int _{{\mathbb {S}}^{d-1}}\hat{\mathbf{v}}\,q(\varvec{\xi },\hat{\mathbf{v}})\int _{{\mathbb {R}}_+}\gamma (\lambda )\,{\mathcal {S}}(\varvec{\xi }+\lambda \hat{\mathbf{v}})\,d\lambda \,d\hat{\mathbf{v}}\,\\&\quad \, +\dfrac{{\bar{U}}}{c_1(\varvec{\xi })}\int _{{\mathbb {S}}^{d-1}} \hat{\mathbf{v}}\otimes \hat{\mathbf{v}}\,\nabla q(\varvec{\xi },\hat{\mathbf{v}}) \int _{{\mathbb {R}}_+}\lambda \,\gamma (\lambda ){\mathcal {S}}(\varvec{\xi }+\lambda \hat{\mathbf{v}}) \, d\lambda \, d\hat{\mathbf{v}}\end{aligned} \end{aligned}$$

and, thus, we perform a hyperbolic limit leading to (35). The mean velocity (69) is a linear combination of a non-local measure of the chemoattractant ${\mathcal {S}}$ over the fiber network and a non-local average of ${\mathcal {S}}$ weighted by the directional average of the fiber direction spatial variability.

#### Comments

We can observe that, if $\gamma _q=\gamma _{{\scriptscriptstyle {\mathcal {S}}}}=\gamma =\delta _R(\lambda )$, the two non-local transport models for independent and dependent sensing are equal, while, if the sensing kernels are not Dirac deltas (even if $\gamma _q=\gamma _{{\scriptscriptstyle {\mathcal {S}}}}=\gamma$), the transport models are always different. Instead, at the macroscopic level, with any choice of the sensing functions the models coincide only in case (*ii*), i.e., when we have a slow variation of both cues. In this case, in fact, the macroscopic limits are different only if $\gamma _q \ne \gamma _{{\scriptscriptstyle {\mathcal {S}}}}$, while in the cases (*iii*), i.e., when we have fast *q* and slow ${\mathcal {S}}$ variation and *iv*), i.e., when we have fast ${\mathcal {S}}$ and slow *q* variation, they are different if the sensing kernel are not Dirac deltas (even if $\gamma _{{\scriptscriptstyle {\mathcal {S}}}}=\gamma _q=\gamma$). The relevant difference concerns the macroscopic transport velocities (see (56) and (67) for the case (*iii*) and (58) and (69) for the case (*iv*). In fact, in the cases (*iii*) and (*iv*), for the non-local dependent sensing model, since only one cue is considered non-locally and both cues are averaged with the same sensing function $\gamma$, we have a weighted average on $\lambda$ of the non-local quantities, which results in the weighted averages in the second terms of (67) and (69). Table 1 presents a general summary of all the models we derived, while we summarized these remarks in Table 2.

**Table 1:** Summary of the models (dropping the local dependencies in $\varvec{\xi }$)

Case	Non-local independent sensing (4)-(5)-(14)	Non-local dependent sensing (4)-(5)-(15)
*i*) *fast* ${\mathcal {S}}$ *and slow q*	Drift dominated	Drift dominated
	$\mathbf{U}_T= c{\bar{U}}\!\!\displaystyle \int _{{\mathbb {S}}^{d-1}}\!\!\!\hat{\mathbf{v}}\!\!\displaystyle \int _{ 0}^{ R}\!\!\!\!\gamma _{{\mathcal {S}}}(\lambda ) {\mathcal {S}}(\varvec{\xi }+\lambda \hat{\mathbf{v}})d\lambda \!\!\int _{ 0}^{ R}\!\!\!\!\gamma _q(\lambda )\,q(\varvec{\xi }+\lambda \hat{\mathbf{v}},\hat{\mathbf{v}})d\lambda d\hat{\mathbf{v}}$	$\mathbf{U}_T=c{\bar{U}}\!\!\displaystyle \int _{{\mathbb {S}}^{ d-1}}\!\!\hat{\mathbf{v}}\!\!\int _{0}^R\!\!\!\!\gamma (\lambda ) {\mathcal {S}}(\varvec{\xi }+\lambda \hat{\mathbf{v}}) q(\varvec{\xi }+\lambda \hat{\mathbf{v}},\hat{\mathbf{v}})d\lambda \,d\hat{\mathbf{v}}$
*ii*) *slow* ${\mathcal {S}}$ *and q*	Drift-diffusion	Drift-diffusion
	${\mathbb {D}}_T^0=D\,{\mathbb {D}}_q$	${\mathbb {D}}_T^0=D\,{\mathbb {D}}_q$
	$\mathbf{U}_T^1={\bar{U}}\,\left[ \Gamma ^q\,\nabla \cdot {\mathbb {D}}_q+\,\Gamma ^{\tiny {\mathcal {S}}}\,{\mathbb {D}}_q\,\dfrac{\nabla {\mathcal {S}}}{{\mathcal {S}}}\right]$	$\mathbf{U}_T^1={\bar{U}}\Gamma \,\left[ \nabla \cdot {\mathbb {D}}_q+\,{\mathbb {D}}_q\,\,\dfrac{\nabla {\mathcal {S}}}{{\mathcal {S}}}\right]$
*iii*) *fast* *q* *and slow* ${\mathcal {S}}$	Drift dominated	Drift dominated
	$\mathbf{U}_T=\dfrac{{\bar{U}}\,\Gamma _0^{\mathcal {S}}}{c_0}\,{\mathcal {S}}\displaystyle \int _{{\mathbb {R}}_+}\gamma _q(\lambda )\,\int _{{\mathbb {S}}^{d-1}}\hat{\mathbf{v}}\,q(\varvec{\xi }+\lambda \hat{\mathbf{v}},\hat{\mathbf{v}})\,d\hat{\mathbf{v}}\,d\lambda$	$\mathbf{U}_T:=\dfrac{{\bar{U}}}{c_0}\displaystyle \int _{{\mathbb {R}}_+}\gamma (\lambda )\,\int _{{\mathbb {S}}^{d-1}}\hat{\mathbf{v}}q(\varvec{\xi }\,+\lambda \hat{\mathbf{v}},\hat{\mathbf{v}})\,d\hat{\mathbf{v}}\,d\lambda$
	$\quad \qquad +\dfrac{{\bar{U}}\,\Gamma _1^{\mathcal {S}}}{c_1} \nabla {\mathcal {S}}\,\displaystyle \int _{{\mathbb {R}}_+}\gamma _q(\lambda )\,\displaystyle \int _{{\mathbb {S}}^{d-1}}\hat{\mathbf{v}}\otimes \hat{\mathbf{v}}\,q(\varvec{\xi }+\lambda \hat{\mathbf{v}},\hat{\mathbf{v}}) \,d\hat{\mathbf{v}}\,d\lambda$	$\quad \qquad \quad + \dfrac{{\bar{U}}}{c_1}\nabla {\mathcal {S}}\displaystyle \int _{{\mathbb {R}}_+}\lambda \, \gamma (\lambda )\,\int _{{\mathbb {S}}^{d-1}}q(\varvec{\xi }+\lambda \hat{\mathbf{v}},\hat{\mathbf{v}})\hat{\mathbf{v}}\otimes \hat{\mathbf{v}}\,d\hat{\mathbf{v}}\,d\lambda$
*iv*) *fast* ${\mathcal {S}}$ *and slow* *q*	Drift dominated	Drift dominated
	$\mathbf{U}_T=\dfrac{{\bar{U}}\,\Gamma _0^q}{c_0}\,\displaystyle \int _{{\mathbb {S}}^{d-1}}\!\!\!\hat{\mathbf{v}}\,q\,\,\int _{0}^R\!\!\!\!\gamma _{\tiny {\mathcal {S}}}(\lambda )\,{\mathcal {S}}(\varvec{\xi }+\lambda \hat{\mathbf{v}})\,d\lambda \,d\hat{\mathbf{v}}$	$\mathbf{U}_T:=\dfrac{{\bar{U}}}{c_0}\displaystyle \int _{{\mathbb {S}}^{d-1}}\!\!\!\hat{\mathbf{v}}\,q\int _{0}^R\!\!\!\!\gamma (\lambda )\,{\mathcal {S}}(\varvec{\xi }+\lambda \hat{\mathbf{v}})\,d\lambda \,d\hat{\mathbf{v}}\,$
	$\quad \qquad +\dfrac{{\bar{U}}\,\Gamma _1^q}{c_1}\displaystyle \int _{{\mathbb {S}}^{d-1}}\!\!\!\hat{\mathbf{v}}\otimes \hat{\mathbf{v}}\,\nabla q \int _{0}^R\!\!\!\!\gamma _{{\scriptscriptstyle {\mathcal {S}}}}(\lambda )\,{\mathcal {S}}(\varvec{\xi }+\lambda \hat{\mathbf{v}}) \, d\lambda \, d\hat{\mathbf{v}}$	$\quad \qquad \quad +\dfrac{{\bar{U}}}{c_1}\displaystyle \int _{{\mathbb {S}}^{d-1}} \!\!\!\hat{\mathbf{v}}\otimes \hat{\mathbf{v}}\,\nabla q \int _{0}^R\!\!\!\!\lambda \,\gamma (\lambda ){\mathcal {S}}(\varvec{\xi }+\lambda \hat{\mathbf{v}}) \, d\lambda \, d\hat{\mathbf{v}}$

**Table 2:** Summary of the comparison of the models for different choices of the sensing functions. $\checkmark$ indicates the cases in which the models coincide, while  the ones in which the models are different

	$\gamma _q=\gamma _{{\scriptscriptstyle {\mathcal {S}}}}=\gamma =\delta$	$\gamma _q=\gamma _{{\scriptscriptstyle {\mathcal {S}}}}=\gamma \ne \delta$	$\gamma _q\ne \gamma _{{\scriptscriptstyle {\mathcal {S}}}}$
Meso models (4)-(5)-(14) and (4)-(5)-(15)	$\checkmark$		
Macro models case (*i*): *fast* ${\mathcal {S}}$ *and* *q*	$\checkmark$		
Macro models case (*ii*): *slow* ${\mathcal {S}}$ *and* *q*	$\checkmark$	$\checkmark$	
Macro models case (*iii*): *fast* *q* *and slow* ${\mathcal {S}}$	$\checkmark$		
Macro models case (*iv*): *fast* ${\mathcal {S}}$ *and slow* *q*	$\checkmark$		

## Numerical Simulations

We now present simulations of the kinetic transport model (4)-(5) for non-local independent sensing (14) and non-local dependent sensing (15) in order to show some key model features. Precisely, we numerically integrate the transport equation to approximate the density distribution *p*, as in Filbet and Yang (2014); Loy and Preziosi (2020), and in turn the corresponding macroscopic density via (1). For computational convenience, we restrict to a rectangular 2D region $\Omega = [0, 5] \times [0, 5]$.

We specify *q* using the standard circular distribution given by the bimodal von Mises distribution (Mardia and Jupp 2009)

70 $$\begin{aligned} q\left( \mathbf{x},\hat{\mathbf{v}}\right) =\dfrac{1}{4\pi I_0(k(\mathbf{x}))}\left( e^{k(\mathbf{x})\,\mathbf{u}(\mathbf{x})\cdot \hat{\mathbf{v}}}+e^{-k(\mathbf{x})\,\mathbf{u}(\mathbf{x})\cdot \hat{\mathbf{v}}}\right) , \end{aligned}$$

where $I_\nu (k)$ is the modified Bessel function of first kind of order $\nu$ and

$$\begin{aligned} \mathbf{u}(\mathbf{x})=(\cos (\theta _q(\mathbf{x})),\sin (\theta _q(\mathbf{x}))). \end{aligned}$$

It can be proved that the first trigonometric moment is $\mathbf{E}_{q}(\mathbf{x})=\mathbf{u}(\mathbf{x})$ (Hillen et al. 2017), and, therefore, $\theta _q(\mathbf{x})$ is the mean direction of the fibers located at point $\mathbf{x}$ (Mardia and Jupp 2009). This function also satisfies Q3 and its variance–covariance matrix is given by Hillen et al. (2017)

$$\begin{aligned} {\mathbb {D}}_q(x)=\dfrac{1}{2}\left( 1-\dfrac{I_2(k)}{I_0(k)} \right) {\mathbb {I}}_2+\dfrac{I_2(k)}{I_0(k)}\mathbf{u}\otimes \mathbf{u}, \end{aligned}$$

where ${\mathbb {I}}_2$ is the identity tensor in ${\mathbb {R}}^{2\times 2}$, while *k* and $\mathbf{u}$ are functions of $\mathbf{x}$. Moreover, the circular-variance is given by the scalar

$$\begin{aligned} D_q(\mathbf{x})=\left( 1-\dfrac{I_1(k)}{I_0(k)}\right) \end{aligned}$$

that represents the degree of alignment of the fibers at point $\mathbf{x}$.

We propose three sets of numerical tests in which we integrate the kinetic transport equation (4) and visualize the macroscopic density (1):

**Test 1:** a preliminary test for showing the effects of a non-local chemoattractant in the presence of a local fiber network;
**Test 2:** a comparison between the two sensing strategies with different sensing kernels;
**Test 3:** a set of simulations in different scenarios (${ i.e.}$, for different $l_q, l_{{\mathcal {S}}}, R$), allowing to make some observations about the differences between the emerging macroscopic regimes.

### Test 1: Local ECM Sensing and Non-Local Chemotaxis

Test 1 is designed to highlight the effect of the presence of a chemoattractant on the behavior of cells migrating by contact guidance over a non-polarized fiber network and evaluating locally its alignment. This means that for this test we consider $q=q(\mathbf{x},\hat{\mathbf{v}})$. In the absence of additional cues, when fibers are sensed locally, even though the distribution *q* is anisotropic, cells are distributed symmetrically in each given direction as there is no drift induced by an asymmetric external cue (Hillen 2006; Painter 2008). Conversely, in presence of a chemoattractant, if it is sensed non-locally, a chemotactic velocity is imposed. Formally, we are dealing with (14) in which $\gamma _q=\delta _0(\lambda )$. In particular, we consider a region

71 $$\begin{aligned} \Omega _q=\left\{ \mathbf{x}=(x,y)\in \Omega \quad s.t. \quad x_1 \le x \le x_2 \right\} \end{aligned}$$

with $x_1=1.8$ and $x_2=3.2$ in which the fibers are strongly aligned along the direction identified by $\theta _q=\pi /2$. In particular, for $(x,y) \in \Omega _q$, $k(x,y)=700$, such that $D_q=5\cdot 10^{-3}$ . In the rest of the domain $\Omega -\Omega _q$ fibers are uniformly distributed. The chemoattractant has a fixed Gaussian profile

72 $$\begin{aligned} {\mathcal {S}}(x,y)=\dfrac{m_{{\mathcal {S}}}}{\sqrt{2\pi \sigma _{{\mathcal {S}}}^2}}e^{-\dfrac{\left( (x,y)-(x_{{\mathcal {S}}},y_{{\mathcal {S}}})\right) ^2}{2\sigma _{{\mathcal {S}}}^2}}. \end{aligned}$$

**Fig. 1:**
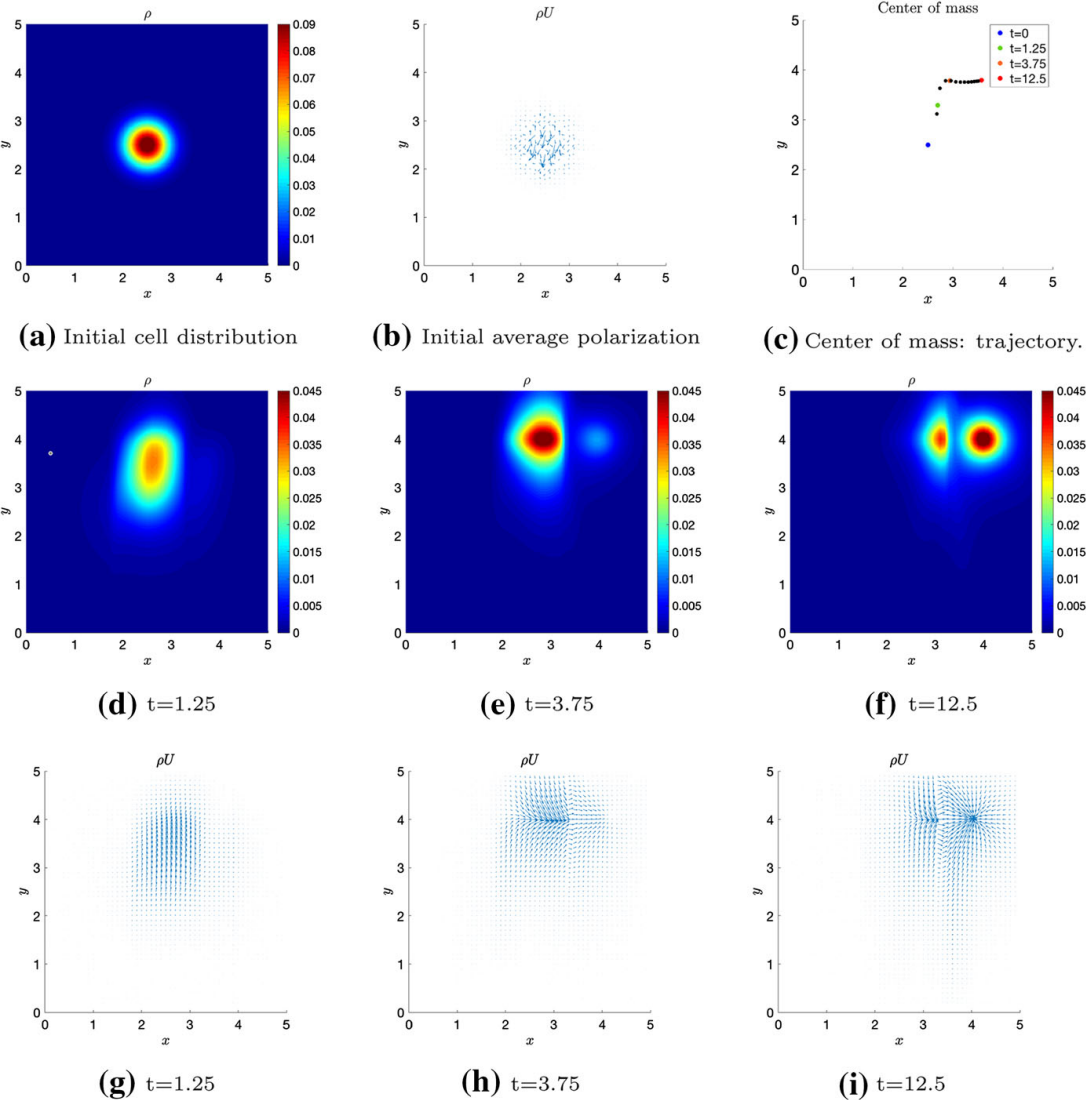
**Test 1**: evolution of the initial cell distribution in (**a**) for local *q* and non-local ${\mathcal {S}}$ with sensing function $\gamma _{{\scriptscriptstyle {\mathcal {S}}}}=\delta _R(\lambda )$. The sensing radius of the cells is set to $R=0.5$, while ${\mathcal {S}}$ is (72) with $m_{{\mathcal {S}}}=10, \sigma _{{\mathcal {S}}}^2=0.05$ and $(x_{\mathcal {S}}, y_{\mathcal {S}})=(4,4)$. (**b**): initial cell orientations. (**c**): trajectory of the center of mass of the cell population, where each black dot is plotted every $\Delta t=1$. (**d**–**f**): evolution of the macroscopic density. (**g**–**i**): polarizations of the cells (color figure online)

In particular, for Test 1 we chose $(x_{{\mathcal {S}}},y_{{\mathcal {S}}})=(4,4), \, m_{{\mathcal {S}}}=10, \sigma _{{\mathcal {S}}}^2=0.1$. The initial condition for the cell population is a Gaussian

73 $$\begin{aligned} \rho _0(x,y)=r_0 e^{-\dfrac{\left( (x,y)-(x_0,y_0) \right) ^2}{2\sigma _0^2}} \end{aligned}$$

with $r_0=0.1$ and $\sigma _0^2=0.1$. In this first test, the initial condition for the cell population is centered in $(x_0,y_0)=(2.5,2.5)$, ${ i.e.}$, the center of the region $\Omega _q$ (see Fig. 1a). We remark that without chemoattractant, cells would diffuse anisotropically in the preferential direction of the fibers $\pm \pi /2$, forming the well known ellipsis (Painter 2008), which represents cells moving with the same probability along directions $\pi /2$ and $-\pi /2$. In the present case, because of the presence of a chemoattractant, the symmetry is broken, and, even if *q* describes a non-polarized fiber network, there is a preferential sense of motion in direction $\pi /2$ (see Fig. 1d–f). Precisely, cells migrate along the fibers in the direction identified by $\theta _q=\pi /2$, corresponding to the preferential sense imposed by the presence of the chemoattractant located in the upper-right corner of the domain $\Omega$. Given this directional setting, the cell population dynamics is also greatly affected by the strength of the chemoattractant, which depends on $m_{{\mathcal {S}}}$ and $\sigma _{{\mathcal {S}}}^2$, by the degree of the alignment $D_q$, which depends on *k*(*x*, *y*), and by the sensing radius *R*.

Another important aspect is the sensing function $\gamma _{{\scriptscriptstyle {\mathcal {S}}}}$, which influences the transient dynamics and, especially, the relaxation time. For example, in the case of a Heaviside function, the relaxation time is twice the relaxation time needed when $\gamma _{{\scriptscriptstyle {\mathcal {S}}}}$ is a Dirac delta (see also Loy and Preziosi 2020). Moreover, we analyzed the average cell polarization at every position $\mathbf{x}$, given by the mass flow (2), that is the average velocity times the macroscopic cell density. The cell microscopic orientations are initially randomly distributed and they start from a vanishing initial speed (see Fig. 1b). Then, they start to orient and align along the fibers, migrating upward in the direction individuated by the angle $\pi /2$, since cells sense the chemoattractant (see Fig. 1g–h). Eventually, when cells reach the level $y=4$, the microscopic directions polarize toward the chemoattractant (see Fig. 1i). The center of mass plotted in Fig. 1c stays in the region $\Omega _q$ during cell migration along the fibers bundle in $\Omega _q$, and it moves out of $\Omega _q$ only when it reaches $y=4$. The black dots are plotted every $\Delta t=1$ and it is clear that the highest acceleration happens when cells are on the bundle of fibers, while they are slowed down when they start to move out of the fibers stripe $\Omega _q$.

### Test 2: Non-Local ECM Sensing and Chemotaxis

**Fig. 2:**
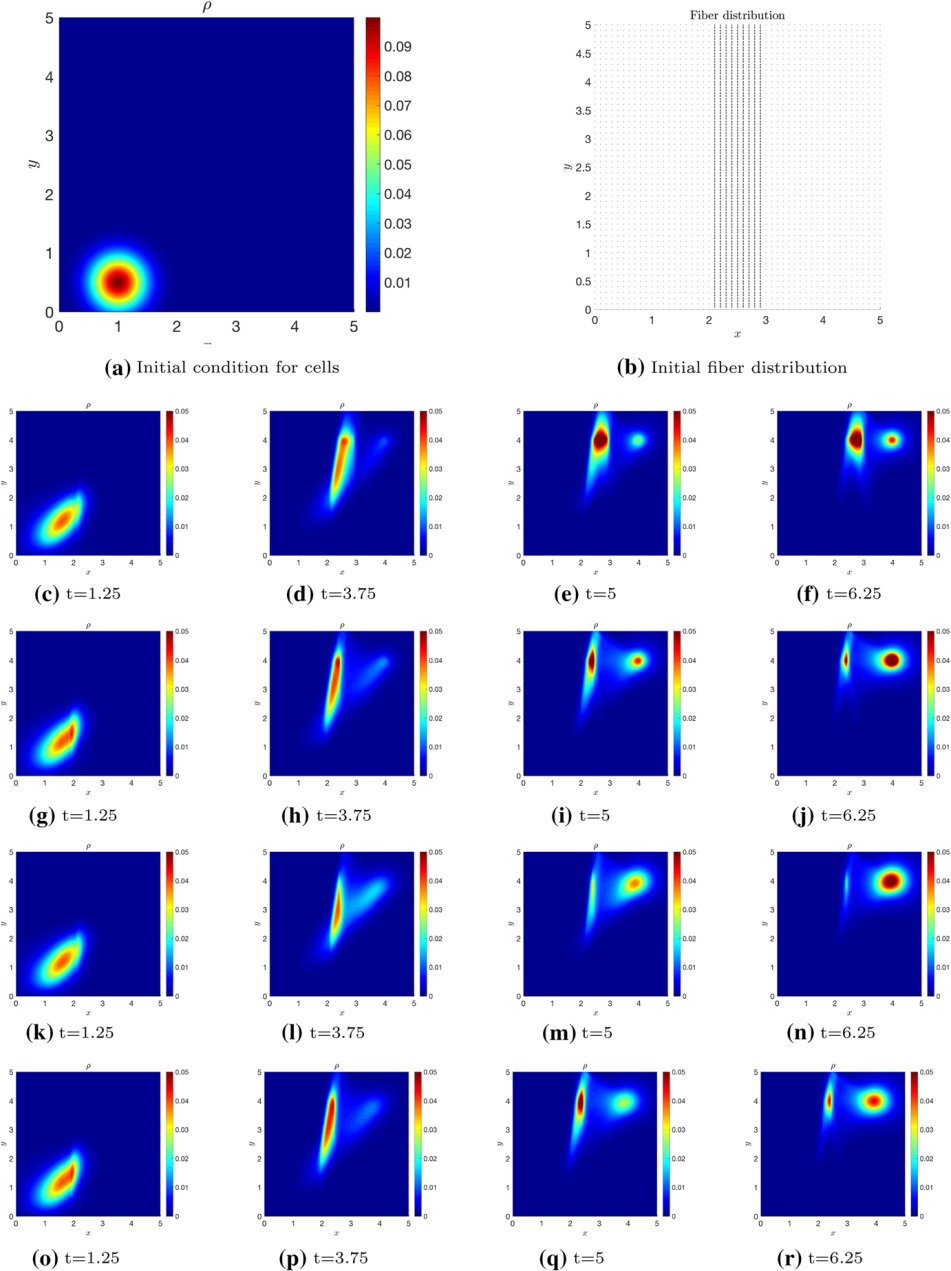
**Test 2** Time evolution of the initial distribution given in Fig. 2a in the four settings 1-4. The sensing radius of the cells is $R=0.5$ and ${\mathcal {S}}$ is (72) with $m_{{\mathcal {S}}}=10, \sigma _{{\mathcal {S}}}^2=0.05$ and $(x_{\mathcal {S}}, y_{\mathcal {S}})=(4,4)$. Setting 1 is represented in Figs. (**c–f**): local *q* and non-local ${\mathcal {S}}$, $\gamma _{{\scriptscriptstyle {\mathcal {S}}}}=\delta _R(\lambda )$. Setting 2 is represented in Figs. (**g–j**): non-local *q* and ${\mathcal {S}}$ with sensing functions $\gamma _q=\gamma _{{\scriptscriptstyle {\mathcal {S}}}}=\delta _R(\lambda )$. Setting 3 is represented in Figs. (**k–n**): non-local *q* and ${\mathcal {S}}$, independent sensing with $\gamma _q=\gamma _{{\scriptscriptstyle {\mathcal {S}}}}=H(R-\lambda )$. Setting 4 is represented in Figs. (**o–r**): non-local *q* and ${\mathcal {S}}$, dependent sensing with $\gamma =H(R-\lambda )$ (color figure online)

In this second test, we compare the non-local independent sensing model and the non-local dependent sensing model. We assume fibers to be distributed similarly to the previous test, ${ i.e.}$, fibers are highly aligned in $\Omega _q$, defined this time by $x_1=2.1$ and $x_2=2.9$ (see Fig. 2b). Here, for $(x,y) \in \Omega _q$, $k(x,y)=100$, that corresponds to $D_q=0.0025$, and $\theta _q(x,y)=\pi /2$. In the region $\Omega -\Omega _q$ fibers are uniformly distributed. The initial condition of the cell population is (73) with $(x_0,y_0)=(1,0.5)$ (see Fig. 2a) while the chemoattractant has a fixed profile located as in Test 1, with $m_{{\mathcal {S}}}=10$ and $\sigma _{{\mathcal {S}}}^2=0.05$. We compare the dynamics of the cell population in the following four settings:

1. local fiber distribution and non-local chemoattractant, as in Test 1, ${ i.e.}$, (14) with $\gamma _q=\delta _0(\lambda )$ and $\gamma _{{\scriptscriptstyle {\mathcal {S}}}}=\delta _R(\lambda )$;
2. non-local sensing with Dirac Deltas for both *q* and ${\mathcal {S}}$, ${ i.e.}$, we integrate (4) with (14) or (15) with $\gamma _q=\gamma _{{\scriptscriptstyle {\mathcal {S}}}}=\gamma =\delta _R(\lambda )$;
3. non-local independent sensing with Heaviside sensing functions for both ${\mathcal {S}}$ and *q*, ${ i.e.}$, (4)-(14) with $\gamma _q=\gamma _{{\scriptscriptstyle {\mathcal {S}}}}=H(R-\lambda )$;
4. non-local dependent sensing with Heaviside sensing function for *q* and ${\mathcal {S}}$, ${ i.e.}$, (4)-(15) with $\gamma =H(R-\lambda )$.

Results of these simulations are shown in Fig. 2.

We can observe that, in all settings, cells start from (1, 0.5), they are attracted by the chemoattractant and, on their way towards ${\mathcal {S}}$, they cross the aligned fibers region $\Omega _q$, climbing up this region in the direction $\pi /2$. Eventually, in all the cases, cells reach the chemoattractant, but the dynamics, as well as the transient time, is influenced by different sensing kernels and the local or non-local sensing strategy, even though the differences are not extremely evident. Although settings 3 and 4 in Fig. 2, which are related to the case of independent and dependent cues, respectively, do not seem to show very strong differences, we can observe that in case 3 (see Fig. 2k–n) the tendency of going in both the directions $\pi /2$, determined by *q*, and $\pi /4$, determined by ${\mathcal {S}}$, appears more marked because of the independent sensing. In contrast, this behavior is less evident in case 4 and it results the least evident in the case in which cells deal with a local sensing of the fibers (setting 1), with also a general slow down of the dynamics.

### Test 3: Non-Local Independent Sensing Model for the Comparison of Cases $(i)-(iv)$

Test 3 is designed to explore the extent to which the macroscopic cell behavior changes depending on the relation between *R*, $l_{\mathcal {S}}$ and $l_q$. Precisely, we compare cases (*i*), (*ii*), (*iii*) and *iv*) according to different mutual variations of the external cues *q* and ${\mathcal {S}}$. The aim here is to illustrate the importance of choosing the most appropriate macroscopic model, as the aggregate cell behavior can be very different according to the regime prescribed by the system parameters. We perform the analysis for the non-local independent sensing case with $\gamma _q=\gamma _{{\scriptscriptstyle {\mathcal {S}}}}=H(R-\lambda )$, as it is a case in which the transport model is different from the dependent sensing model. Moreover, the independence of the two sensing allows to visualize more efficiently the two distinct directional effects (contact guidance and chemotaxis), as observed from the results of Fig. 2.

We consider the turning kernel describing contact guidance with *q* given by (70), $\theta _q(x,y)=3\pi /4$ $\forall (x,y) \in \Omega$, and coefficient *k*(*x*, *y*), modulating the strength of the alignment, given by the gaussian distribution

74 $$\begin{aligned} k(x,y)=m_k e^{-\dfrac{\left( (x,y)-(x_k,y_k)\right) ^2}{2\sigma _k^2}}\,. \end{aligned}$$

**Fig. 3:**
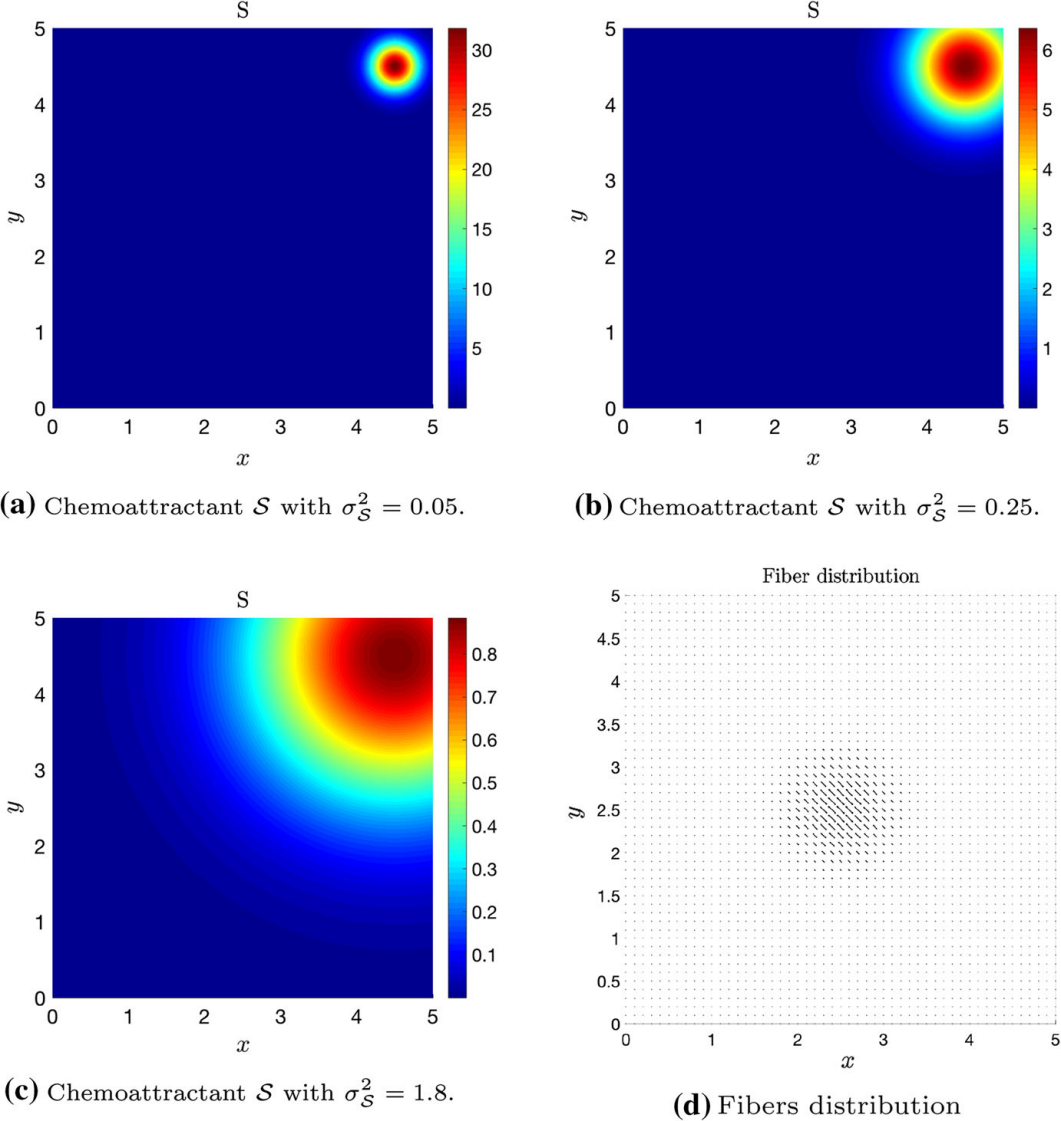
**Test 3** Three different chemoattractant distributions used for comparing models $$i)-iv)$$. The chemoattractant profile is given by (72) with $$m_{{\mathcal {S}}}=10$$ and (**a**) $$\sigma _{{\mathcal {S}}}^2=0.05$$, corresponding to $$l_{{\mathcal {S}}}=0.002$$, (**b**) $$\sigma _{{\mathcal {S}}}^2=0.25$$, corresponding to $$l_{{\mathcal {S}}}=0.055$$, and (**c**) $$\sigma _{{\mathcal {S}}}^2=1.8$$, corresponding to $$l_{{\mathcal {S}}}=0.25$$. The fibers distribution in sketched in (**d**) (color figure online)

**Fig. 4:**
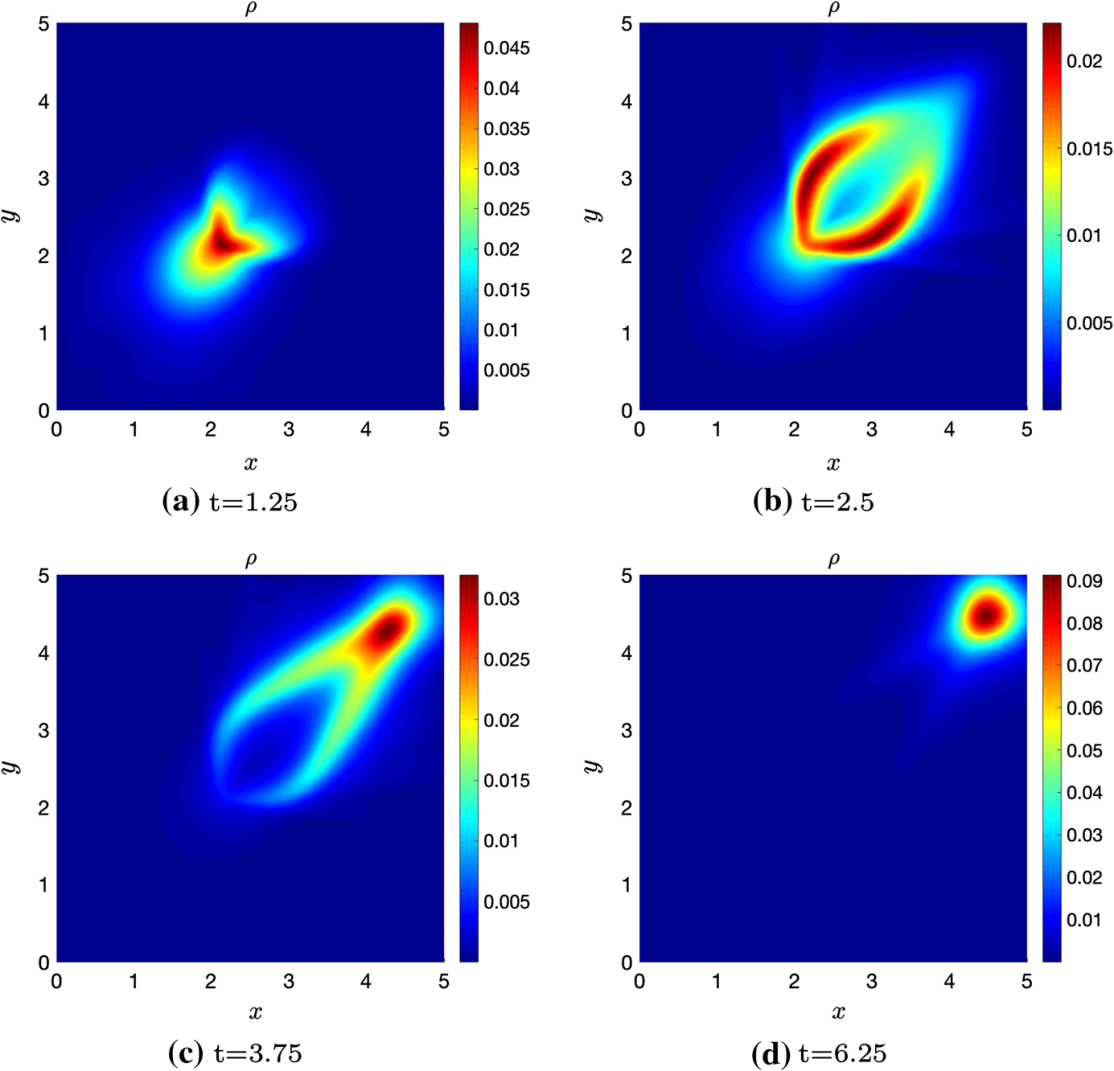
**Test 3** Case (*i*) (fast variation of both cues) with non-local *q* and ${\mathcal {S}}$, sensed with an independent sensing through the kernels $\gamma _q=\gamma _{{\scriptscriptstyle {\mathcal {S}}}}=H(R-\lambda )$. ${\mathcal {S}}$ is given in Fig. 3a with $m_{{\mathcal {S}}}=10$ and $\sigma _{{\mathcal {S}}}^2=0.05$, so that $l_{\mathcal {S}}=0.002$. The fibers distribution *q* has a space dependent parameter *k* given by (74) with $m_{k}=100$, so that $l_q\approx 0.0031$. The sensing radius of the cells is $R=0.7$ (color figure online)

**Fig. 5:**
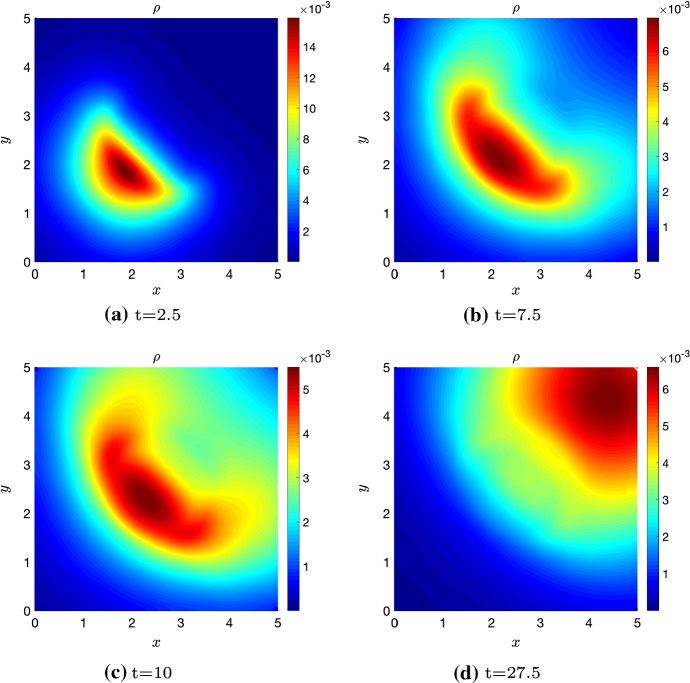
**Test 3** Case (*i*) (fast variation of both cues) with non-local *q* and ${\mathcal {S}}$, independent and sensing with $\gamma _q=\gamma _{{\scriptscriptstyle {\mathcal {S}}}}=H(R-\lambda )$. ${\mathcal {S}}$ is given in Fig. 3c that corresponds to $l_{\mathcal {S}}=0.25$, while for the fiber distribution $m_k=100$, so that $l_q\approx 0.0031$. The sensing radius of the cells is $R=0.7$ (color figure online)

**Fig. 6:**
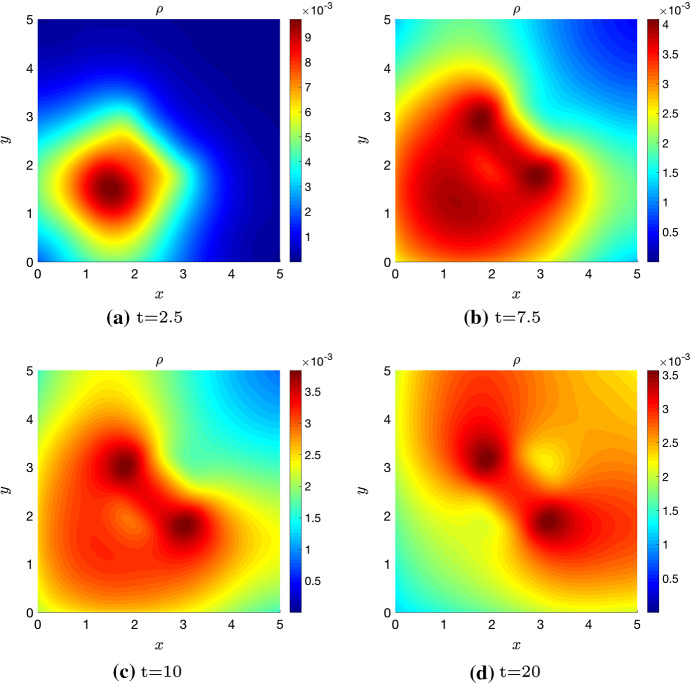
**Test 3** Case (*ii*) (slow variation of both cues) with non-local *q* and ${\mathcal {S}}$, independent and sensing with $\gamma _q=\gamma _{{\scriptscriptstyle {\mathcal {S}}}}=H(R-\lambda )$. ${\mathcal {S}}$ is given in Fig. 3b that corresponds to $l_{\mathcal {S}}=0.055$, while for the fiber distribution $m_k=10$, so that $l_q \approx 0.031$. The sensing radius of the cells is $R=0.02$ (color figure online)

**Fig. 7:**
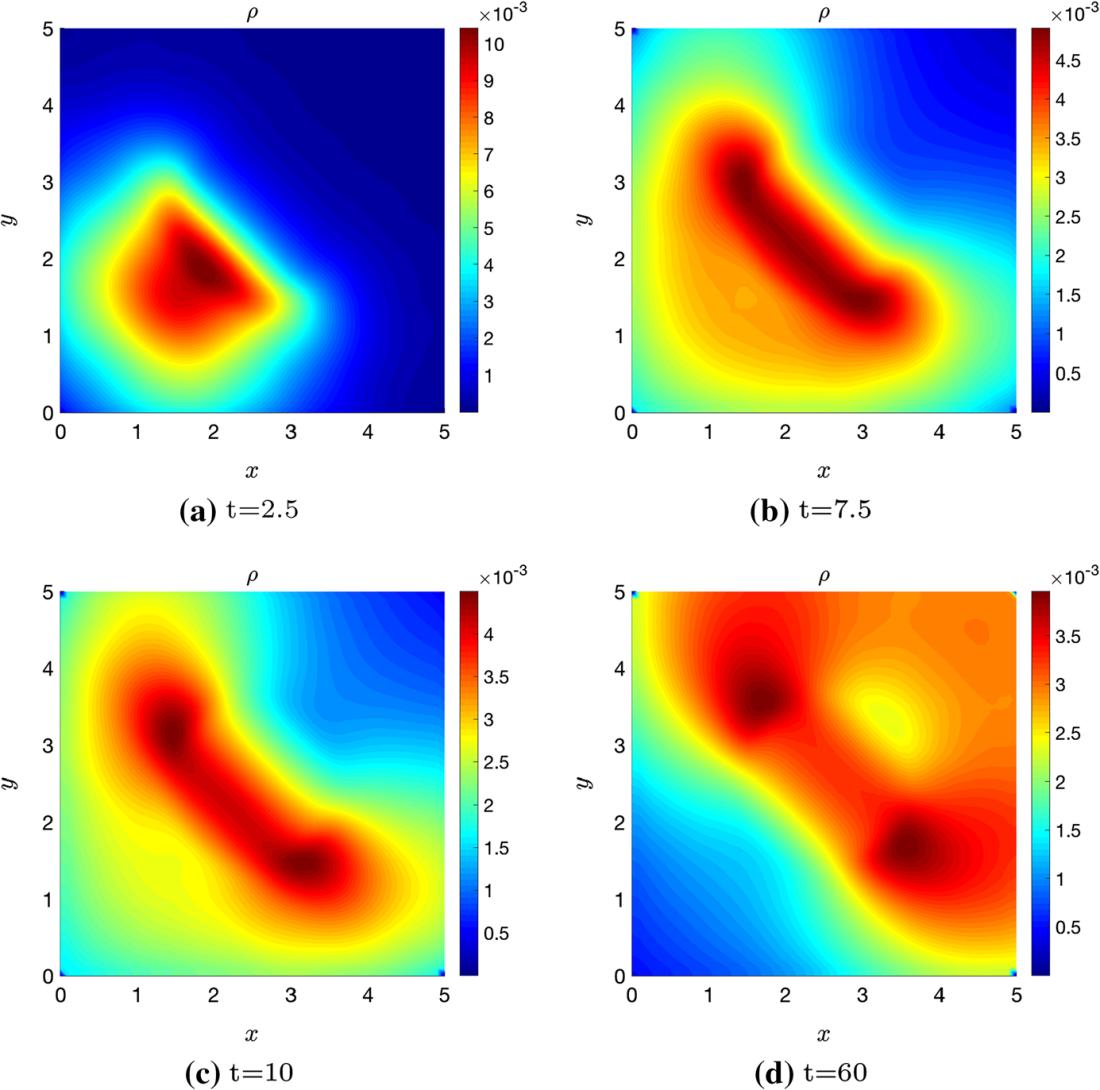
**Test 3** Case (*iii*) (fast *q* and slow ${\mathcal {S}}$ variation) with non-local *q* and ${\mathcal {S}}$, independent and with sensing function $\gamma _q=\gamma _{{\scriptscriptstyle {\mathcal {S}}}}=H(R-\lambda )$. ${\mathcal {S}}$ is given in Fig. 3c, so that $l_{{\mathcal {S}}}=0.25$, while for the fiber distribution $m_k=100$, corresponding to $l_q\approx 0.0031$. The sensing radius of the cells is set to $R=0.2$ (color figure online)

**Fig. 8:**
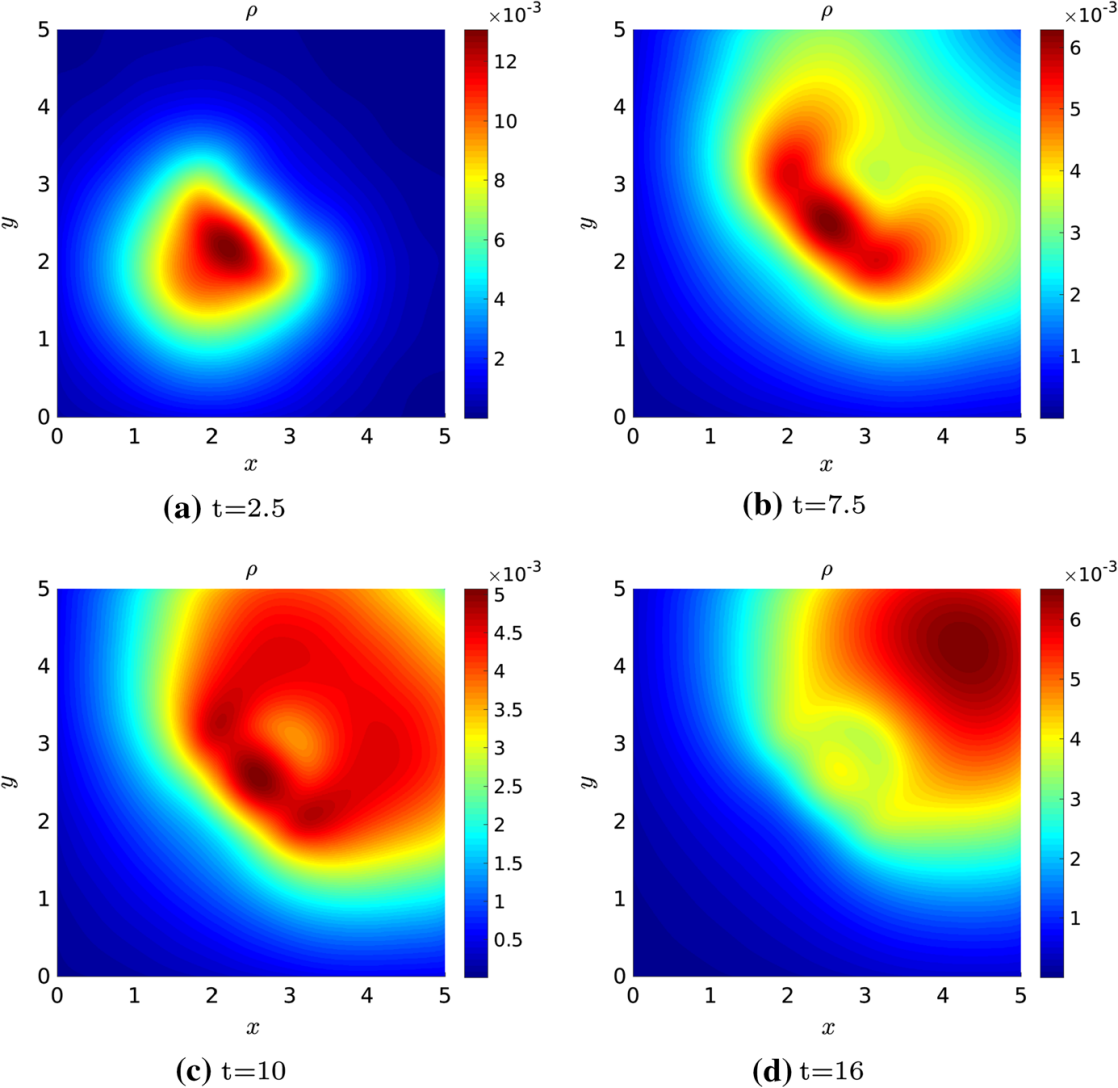
**Test 3** Case (*iv*) (fast ${\mathcal {S}}$ and slow *q* variation) with non-local *q* and ${\mathcal {S}}$, independent sensing with $\gamma _q=\gamma _{{\scriptscriptstyle {\mathcal {S}}}}=H(R-\lambda )$. ${\mathcal {S}}$ is given in Fig. 3a that corresponds to $l_{\mathcal {S}}=0.002$, while for the fiber distribution $m_k=10$, so that $l_q \approx 0.031$. The sensing radius of the cells is $R=0.02$ (color figure online)

Here, $(x_k,y_k) = (2.5,2.5)$ and $\sigma _k^2=0.15$ (Fig. 3d). This choice describes a setting in which fibers are more aligned in the central circular region of the domain and uniformly disposed in the rest of it. We consider different values of $m_k$ in order to obtain different values of $l_q$: $m_k=10$ corresponds to $l_q \approx 0.031$ and $m_k=100$ corresponds to $l_q\approx 0.0031$. Details about the estimation of $l_q$ for a Bimodal Von Mises distribution of fibers *q* are given in Appendix A. The chemoattractant has the fixed profile (72) with $(x_{{\mathcal {S}}},y_{{\mathcal {S}}})=(4.5, 4.5)$ and $m_{{\mathcal {S}}}=10$. In the simulations, we consider three different values for the chemoattractant variance $\sigma _{{\mathcal {S}}}^2$ in order to obtain different values of $l_{{\mathcal {S}}}$: $\sigma _{{\mathcal {S}}}^2=0.05$, which corresponds to $l_{{\mathcal {S}}}=0.002$ in Fig. 3a; $\sigma _{{\mathcal {S}}}^2=0.25$, which corresponds to $l_{{\mathcal {S}}}=0.055$ in Fig. 3b; $\sigma _{{\mathcal {S}}}^2=1.8$, which corresponds to $l_{{\mathcal {S}}}=0.25$ in Fig. 3c. The initial cell distribution for all the settings presented in Figs. 4-8 is given by (73) with $(x_0,y_0)=(1.5,1.5)$, $r_0=0.1$, $\sigma _0^2=0.1$. Precisely, we present five sets of simulations that are summarized in Table 3.

In Fig. 4, we consider the case in which $\eta _{\mathcal {S}},\eta _q\gg 1$, ${ i.e.}$, fast variation of both external cues (case *i*). This corresponds to a strongly hyperbolic macroscopic behavior with macroscopic velocity given by (43). In Fig. 4 we can observe that cell behavior is not diffusive and the cluster of cells is quite compact. Moreover, when cells reach the central region where fibers are strongly aligned in the direction $3\pi /4$ (as shown in Fig. 3d), which is perpendicular to the favorable direction $\pi /4$ induced by ${\mathcal {S}}$, they surround this region and go over it toward ${\mathcal {S}}$. In this setting, the parameter $\eta$ defined in (42) is slightly smaller then 1 and, in fact, chemotaxis prevails in the overall dynamics, as the stationary state is clearly peaked on the chemoattractant profile, but the fibers structure influences the transient.

In Fig. 5, we consider ${\mathcal {S}}$ with $\sigma _{{\mathcal {S}}}^2=1.8$ and, consequently, $l_{{\mathcal {S}}}=0.25$ (see Fig. 3c). Concerning the fibers, we have $m_k=100$, so that $l_q \approx 0.0031$, and the sensing radius is $R=0.7$. This setting falls again in case (*i*), ${ i.e.}$ fast variation of both cues, but the behavior is different with respect to the previous simulation in Fig. 4. The chemoattractant in Fig. 3c, in fact, is spread over the whole domain and, actually, the quantity $l_{{\mathcal {S}}}$ is almost $10^2$ times the $l_{{\mathcal {S}}}$ considered in Fig. 3a (and used for the simulation in Fig. 4). Even though we are still in a hyperbolic case and cells are guided by the strong drift (43), as *R* is slightly larger than $l_{{\mathcal {S}}}$ and $l_{{\mathcal {S}}}$ is large, the cell cluster diffuses a bit more in the domain. When cells reach the region of strongly aligned fibers, they start to surround it (see Fig. 5a–c), but, as $\eta _{{\mathcal {S}}}= 2.8={\mathcal {O}}(1)$, some of them do not surround the region, slow down and partially tend to align along the fibers. In Fig. 5c, for instance, we have a high density of cells in the strongly aligned fiber region. Eventually, cells manage to overcome the area of highly aligned fibers and they tend to converge to the chemoattractant profile (see Fig. 5d). In this setting, the overall dynamics is greatly affected by the fibers and, in fact, $\eta \gg 1$.

The second scenario, illustrated in Fig. 6, refers to case (*ii*), ${ i.e.}$ slow variation of both cues, since the sensing radius $R=0.02$ is smaller than both $l_{\mathcal {S}}=0.055$ and $l_q\approx 0.031$. At the macroscopic level, the behavior of the system is described by the diffusion–advection equation (50) with macroscopic velocity (49). Actually, in Fig. 6 we can observe a highly diffusive behavior, as the cell macroscopic density has invaded almost half of the domain before even starting to feel the influence of the fibers. If we compare the same time step in Figs. 5b and 6b, we see how cells are reaching in both cases the fibers and feeling the region in which fibers are aligned the most. However, in Fig. 5b the cell cluster is much more compact than in Fig. 6b, where, instead, cells already occupied half of the domain, because of diffusion, and we have a high density of cells both in the region that is close to the strongly aligned fiber region and around the initial position. Then, cells start surrounding the central region of strongly aligned fibers, because they already sense the chemoattractant, and, once they have overcome this area, they tend to the chemoattractant profile (see Fig. 6b–d). In particular, in the transient time, cells accumulate the most at the sides of the region with highly aligned fibers. In this specific setting, $\eta >1$ and, in fact, contact guidance highly affects the dynamics.

The third scenario, illustrated in Fig. 7, refers to the case *iii*) describing fast *q* and slow ${\mathcal {S}}$ variation, since the sensing radius $R=0.02$ is smaller than $l_{{\mathcal {S}}}=0.25$, but it is larger than $l_q\approx 0.0031$. The macroscopic setting is described by a drift dominated equation with drift velocity given by (56). As $\eta _{{\mathcal {S}}}<1$, we have that the chemoattractant induces a strong diffusivity, but being $\eta _q >1$, the alignment of fibers strongly affects the dynamics (see Fig. 7c). Comparing, in addition, Figs. 6b and 7b, we have now that the highest cell concentration is in the mean fiber direction $\theta _q=3\pi /4$ in the region surrounding the center of the domain, where the fibers are aligned with a higher degree. As already observed in Sect. 3, this scenario prescribes $\eta \gg 1$ and, in fact, contact guidance dominates again the dynamics.

Eventually, for a sensing radius $R=0.02$ smaller than $l_q \approx 0.031$, but larger than $l_{\mathcal {S}}=0.002$, we are in the case of fast ${\mathcal {S}}$ and slow *q* variation and the macroscopic behavior is approximated by a hyperbolic equation with drift velocity given in (58). Results of the corresponding simulation are presented in Fig. 8. Here, the chemoattractant has the fixed profile shown in Fig. 3a. Cells diffuse in the domain because $\eta _q$ is smaller than 1, and they start moving in a region with randomly disposed fibers (see Fig. 8a). Then, they mainly follow the preferential direction $\pi /4$ thanks to the presence of the chemoattractant. In fact, it induces a strong drift because of the high non-locality, determining $\eta _{{\mathcal {S}}}\gg 1$. Here chemotaxis is slightly dominating the dynamics and, in fact, $\eta <1$.

Lastly, we remark that, due to different parameter settings, the relaxation time toward the final equilibrium configuration is different in the scenarios illustrated in Figs. 4-8. Thus, in each figure, we reported the same intermediate time steps $t=2.5,7.5,10$ for all the presented scenarios (corresponding to the subfigures (a)–(c)) and a different final time step (corresponding to the subfigure (d)) in order to catch the final equilibrium configuration. Precisely, we observe that in Figs. 4 and 8 the time variation is much faster with respect to the other cases. In these scenarios, in fact, we have $\eta <1$, i.e., chemotaxis is prevailing and cells move faster toward the equilibrium configuration. Instead, in Figs.  5-7 we have $\eta >1$, which means a stronger influence of the contact guidance phenomenon. In particular, the fiber orientation is competing with the preferential direction of movement given by the chemoattractant and cells dynamics are slowed down, leading to much larger relaxation times. For better clarity, videos of the simulations of Test 3 are included as Supplementary material.

## Application: Cell Motility on a System of Electrospun Fibers Under a Gradient of VEGFs

As it was mentioned in Introduction, an important aspect related to the study of multi-cue environments concerns the design of engineered scaffolds allowing to direct cell migration by multiple directional cues. In fact, understanding the controlling factors of cell migration and the interplay between them is necessary for designing implants with optimal cellular infiltration and implant integration with native tissue. Moreover, it is important for performing experiments related to phenomena that occur *in vivo* in physiological and pathological processes. In this section, we aim at showing how our theoretical framework for the description of a double cue environment guiding cell orientation is actually able to replicate the experimental results presented by Sundararaghavan et al. (2013). In this work, the authors consider a model system of electrospun hyaluronic acid fibers, which is an engineered scaffold that is able to mimic native tissue and have the control over fiber orientation. On this scaffold, they evaluate the motility of human umbilical vein endothelial cells (HUVECs) considering the cases of aligned and non-aligned fibers of hyaluronic acid in the presence of a gradient of vascular endothelial growth factors (VEGFs). This setting reproduces a quite common biological situation. Endothelial cell migration towards increasing gradients of VEGFs, in fact, is a well-known characteristics of the process of tumor angiogenesis. Especially in hypoxic situation (*i.e.*, when there is a lack of oxygen in the tumor microenvironment), tumor cells are known to produce and release growth factors in the environment in order to attract endothelial cells and start the process of blood vessel formation or remodeling (Onishi et al. 2011). When the underlying tissue on which endothelial cells are migrating is characterized by aligned fibers, the chemotactic cue given by the VEGF gradient has to be integrated with the guidance response to the fiber network. This is, for instance, the case of brain tumors, where the endothelial cell response to tumor-produced VEGF gradients is influenced by the strong alignment of the fiber structure characterizing the brain tissue (e.g., see Lamalice et al. (2007) and reference therein).

**Table 3:** Summary of the simulations presented in Test 3

$l_{{\mathcal {S}}}$	$l_{q}$	*R*	Case	$\eta$	Figs.
0.002	0.0031	0.7	(*i*)	$<1$	4
0.25	0.0031	0.7	(*i*)	$\gg 1$	5
0.055	0.031	0.02	(*ii*)	$>1$	6
0.25	0.0031	0.2	(*iii*)	$\gg 1$	7
0.002	0.031	0.02	(*iv*)	$<1$	8

**Fig. 9:**
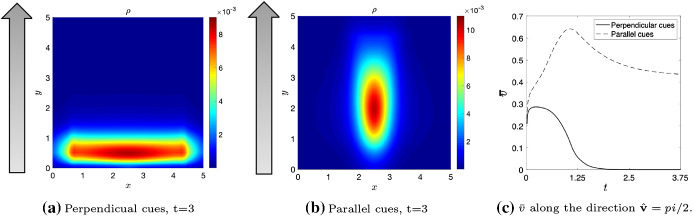
Application: migration of cells on a system of electrospun fibers under a VEGF gradient. Cell macroscopic density at time $t=3$ in response to a chemotactic gradient oriented toward the north (as indicated by the arrow) is illustrated in two scenarios: fibers horizontally oriented in the entire domain (**a**) and fibers vertically oriented in the entire domain (**b**). The cell sensing radius in both cases is $R=0.7$. The time evolution of the cell mean speed defined in (75) is shown in (**c**) for the case of perpendicular cues (continuous line that corresponds to scenario (**a**)) and parallel cues (dashed line that corresponds to scenario (**b**))(color figure online)

In this section, we want to show how our model is able to recover different behaviors of the cells in response to two main experimental settings: fibers parallelly aligned to the direction of the chemotactic gradient or fibers perpendicularly aligned to the chemotactic gradient. Precisely, we would like to capture the cell dynamics shown in Fig. 3C and D of Sundararaghavan et al. (2013). As initial distribution of cells, we consider (73) with $(x_0,y_0)=(2.5,0.5)$, $r_0=0.1$, and $\sigma _0^2=0.05$, while the chemoattractant is given by the linear function

$$\begin{aligned} {\mathcal {S}}(x,y)=m_{{\mathcal {S}}}y \end{aligned}$$

with $m_{{\mathcal {S}}}=10$. The fibers are described with (70) and they are strongly aligned along the direction identified by $\theta _q=\pi$ in the case represented in Fig. 9a, while they are strongly aligned along the direction given by $\theta _q=\pi /2$ in the case in Fig. 9b. In particular, in both cases, $k(x,y)=100$, so that $D_q=0.0025$. Moreover, in both scenarios we quantify the cell mean speed along the direction $\hat{\mathbf{v}}=\pi /2$ as

75 $$\begin{aligned} {\bar{v}}(t):=\left| \left| \int _0^Uvp(t,\mathbf{x},v,\hat{\mathbf{v}})dv \right| \right| _{L^1_{\vartheta }(\Omega )}\,. \end{aligned}$$

Here, $\Omega :=[0,5]\times [0,5]$ represents the physical space computational domain and $L^1_{\vartheta }(\Omega )$ is the weighted $L^1$-space with weight function $\vartheta :=\dfrac{1}{|\Omega |}$.

Figure 9 shows the resulting cell behavior in the two scenarios. In particular, in Fig. 9a, we consider the case of perpendicular cues, meaning that the angle between the mean fiber orientation and the chemotactic gradient direction is $\pi /2$. In this case, the competition between the two cues is evident. In fact, due to the strength of the fiber alignment, cells starting from their initial gaussian distribution align along the fiber direction without being able to move toward the chemoattractant gradient. This is in line with the results shown in Fig. 3C of Sundararaghavan et al. (2013), where cells are seen to move along the direction of fiber alignment with apparently no influence of the VEGF gradient. Looking at the mean speed evolution in Fig. 9c (continuous line), we observe that the cell mean speed along direction $\pi /2$ rapidly decreases toward zero, meaning that the migration of cells in the direction of the increasing chemotactic gradient is hampered. In Fig. 9b, instead, we analyze the case of parallel cues, i.e., the angle between the mean fiber orientation and the chemotactic gradient direction is 0. The cooperation between the two cues here is evident. In fact cells rapidly migrate following the aligned fibers toward the regions of greater chemoattractant concentration. The numerical results are in a good agreement with the experimental ones, shown in Fig. 3D of Sundararaghavan et al. (2013), where a bias of cells moving up the gradient and along the fiber orientation is observed. Concerning the time evolution of the cell mean speed along direction $\pi /2$ (dashed line in Fig. 9c), there is a fast increase of ${\bar{v}}$ as long as cells are moving toward the upper part of the domain where there is the highest computation of the chemoattractant. Once cells have reached the upper border, the mean speed does not increase anymore. In fact, as an effect of the boundary conditions implemented in the simulations and the non-locality (through the cell sensing radius), cells will stop and accumulate in the region of greater chemoattractant.

## Conclusion

In this work, we have proposed a kinetic model for describing cell migration in a multi-cue environment. Moreover, we have considered that cells perform a non-local sensing of the environment up to a distance *R* (named the sensing radius) from its nucleus. Concerning the environmental stimuli, in the present model there are two guidance cues affecting cell polarization, and, thus, cell direction of motion: contact guidance, that is, a bi-directional cue, and a chemical gradient, that is, a mono-directional cue. We remark that, to the best of our knowledge, this is the first time that a non-local sensing in the physical space of the mesoscopic distribution of fibers is considered.

We introduced two novel classes of models: in the first one, cells perform an independent sensing of the fibers and of the chemical in their neighborhood, while, in the second one, cells average the chemical and the fibers with the same sensing kernel. In the two cases, a particular attention was devoted to the identification of the proper macroscopic limit according to the properties of the turning operator. In presence of a heterogeneous network of fibers and chemical, we detected two parameters, $\eta _q$ and $\eta _{{\mathcal {S}}}$, that measure the relation between the cell sensing radius (*R*) and the characteristic lengths of variation ($l_{{\mathcal {S}}}$ and $l_q$) of the two cues. These parameters allow to discriminate between a diffusion-driven regime with an advective correction and a drift-driven regime. Precisely, when the sensing radius does not exceed the characteristic length of the cues, the bi-directional nature of the fiber network prescribes a diffusive regime, otherwise the hyperbolic scaling leads to a macroscopic drift driven regime. We also defined a new parameter $\eta =l_{\mathcal {S}}/l_q$ that is independent on cell size or sensing capabilities and quantifies the relative contribution of contact guidance to chemotaxis. It provides a first separation between the cases of fiber-dominating and chemotaxis-dominating dynamics ($\eta \gg 1$ or $\eta \ll 1$, respectively). A common feature we noticed in different cases is the resulting dependency of the macroscopic velocity on both the fiber network and the chemoattractant. This aspect enhances the non-trivial influence of contact guidance on the cell drift and this interdependence is in accordance with the model proposed by Wagle and Tranquillo (2000). Moreover, in absence of the chemoattractant, the fiber impact on the drift term could persist for spatial heterogeneous fiber distributions, in accordance to what is observed by Hillen (2006). This feature represents a step forward with respect to Wagle and Tranquillo (2000), in which the drift is a function of contact guidance only through to the presence of a chemical gradient (meaning that, without chemoattractant, there would be no drift).

The numerical simulations of the transport equations pointed out the main features characterizing the two classes of models and the possible scenarios that they are able to capture. We observed that the presence of two cues influencing cell polarization, even when the fibers are sensed locally (Test 1), ensures a preferential sense of motion for cells laying on regions of highly aligned non-oriented fibers, and the non-locality enhances this behavior (Test 2). Moreover, these non-local aspects brings a further level of detail to our model, allowing to obtain different macroscopic behaviors depending on the characteristics of the two sensing (Test 3). We did not observe remarkable differences between the independent and the dependent sensing models, when we assume in the former the same sensing kernel for fibers and chemoattractant (${ i.e.}$, when $\gamma _q=\gamma _{{\scriptscriptstyle {\mathcal {S}}}}$). However, if there are biological observations sustaining the possibility that a cell might implement different strategies for sensing the fibers and the chemoattractant, it would be possible to use our framework (in its independent sensing version) to investigate this scenario. This could also allow to compare the possible outcomes of different sensing approaches with the case of a unique and common sensing strategy. Moreover, Test 3 showed the importance of deriving macroscopic equations from the underlying microscopic dynamics and in the appropriate regime: in fact, a directly postulated drift-diffusion equation would not capture the exact dynamics in all the possible regimes.

Overall, the numerical results highlight how the competitive or collaborative effects of the cues depend, in a first instance, on the angle between their relative orientations, ${ i.e.}$, between the direction of fiber alignment $\theta _q$ and the chemotactic gradient. We observed how there is not a simple additivity effect of the two cues and, especially for the case of competitive cues, the determination of the dominant cue strongly depends on their relative strengths, expressed in terms of both concentration and intensity (degree of alignment of the fiber $k(\mathbf{x})$ and steepness of the chemotactic gradient). Potentially, the case of competitive cues, combined with the non-local aspects of the model, could lead to interesting further analysis, especially concerning the possible effects of a multi-cue environment on cell adhesion or on collective migration processes.

Eventually, we presented an application of our framework, showing its ability to qualitatively reproduce the experimental results obtained by Sundararaghavan et al. (2013) on competition/collaboration between fibers and chemicals. The presented results suggest the potential applicability of our model to explore further settings, especially thanks to its flexibility in being adapted to the different scenarios. Moreover, its applicability relates to the fact that the presented framework can be used to calculate parameters which quantify directed cell migration (e.g., mean square displacement, persistence time, directional persistence, or mean speed Othmer et al. 1988).

We remark that, even if simulations were performed in a two dimensional setting, the transport models (and their macroscopic limits, as a consequence) are formulated in a general d-dimensional setting. Hence, a possible future development is to perform simulations in the three dimensional case that would be much more realistic for mimicking in-vivo migration of cells in the extracellular matrix. Moreover, our framework is flexible and can be adapted to describe other directional cues that might relate, among others, to haptotactic, durotactic or electrotactic mechanisms. In the same spirit as in Loy and Preziosi (2020), we plan to enrich this framework considering a non-constant sensing radius, as it may vary according to the spatial and directional variability of the external guidance cues. Lastly, this study was restricted to the case in which the cues affect only cell polarization, considering a uniform distribution of the speeds. However, in line with Loy and Preziosi (2020, 2020), this setting may be modified to model a multi-cue environment in which the signals also affect the speed of the cells.

### Supplementary Information

Below is the link to the electronic supplementary material.

Supplementary file 1 (mp4 365 KB)

Supplementary file 2 (mp4 1251 KB)

Supplementary file 3 (mp4 6567 KB)

Supplementary file 4 (mp4 2222 KB)

Supplementary file 5 (mp4 5154 KB)

## Estimation of $l_q$

Let us consider the fiber density distribution $q(\mathbf{x},\hat{\mathbf{v}})$ defined by a bimodal Von Mises Fisher

$$\begin{aligned} q(\mathbf{x},\hat{\mathbf{v}})=\dfrac{1}{4\pi I_0(k(\mathbf{x}))}\left( e^{k(\mathbf{x})\,\mathbf{u}\cdot \hat{\mathbf{v}}}+e^{-k(\mathbf{x})\,\mathbf{u}\cdot \hat{\mathbf{v}}}\right) \,, \end{aligned}$$

where $k(\mathbf{x})\in {\mathcal {C}}^1(\Omega )$ and $I_\nu (k(\mathbf{x}))$ denotes the modified Bessel function of first kind of order $\nu$.

We now want to give an estimation for the range of variability of the characteristic length $l_q$, defined as:

$$\begin{aligned} l_q:=\dfrac{1}{\max \limits _{\mathbf{x}\in \Omega }\,\max \limits _{\hat{\mathbf{v}}\in {\mathbb {S}}^{d-1}}\frac{|\nabla q \cdot \hat{\mathbf{v}}|}{q}}\,. \end{aligned}$$

Since $\dfrac{\partial I_0}{\partial k}=\dfrac{I_1(k)}{I_0(k)}$, we have that

$$\begin{aligned} \begin{aligned} \nabla q&=\dfrac{\left( e^{k(\mathbf{x})\,\mathbf{u}\cdot \hat{\mathbf{v}}}-e^{-k(\mathbf{x})\,\mathbf{u}\cdot \hat{\mathbf{v}}}\right) }{4 \pi I_0(k(\mathbf{x}))}\,\nabla k\,(\mathbf{u}\cdot \hat{\mathbf{v}})-\dfrac{\left( e^{k(\mathbf{x})\,\mathbf{u}\cdot \hat{\mathbf{v}}}+e^{-k(\mathbf{x})\,\mathbf{u}\cdot \hat{\mathbf{v}}}\right) }{4 \pi I_0^2(k(\mathbf{x}))}\,\dfrac{\partial I_0}{\partial k}\,\nabla k\\&=\dfrac{\left( e^{k(\mathbf{x})\,\mathbf{u}\cdot \hat{\mathbf{v}}}-e^{-k(\mathbf{x})\,\mathbf{u}\cdot \hat{\mathbf{v}}}\right) }{4 \pi I_0(k(\mathbf{x}))}\,\nabla k\,(\mathbf{u}\cdot \hat{\mathbf{v}})-\dfrac{\left( e^{k(\mathbf{x})\,\mathbf{u}\cdot \hat{\mathbf{v}}}+e^{-k(\mathbf{x})\,\mathbf{u}\cdot \hat{\mathbf{v}}}\right) }{4 \pi I_0(k(\mathbf{x}))}\,\dfrac{ I_1(k(\mathbf{x}))}{I_0(k(\mathbf{x}))}\,\nabla k \end{aligned} \end{aligned}$$

Since $q(\mathbf{x},\hat{\mathbf{v}})>0$, we have:

$$\begin{aligned} \begin{aligned} \dfrac{\nabla q \cdot \hat{\mathbf{v}}}{q} =\left| \dfrac{\left( e^{k(\mathbf{x})\,\mathbf{u}\cdot \hat{\mathbf{v}}}-e^{-k(\mathbf{x})\,\mathbf{u}\cdot \hat{\mathbf{v}}}\right) }{\left( e^{k(\mathbf{x})\,\mathbf{u}\cdot \hat{\mathbf{v}}}+e^{-k(\mathbf{x})\,\mathbf{u}\cdot \hat{\mathbf{v}}}\right) }\,(\mathbf{u}\cdot \hat{\mathbf{v}})-\,\dfrac{ I_1(k(\mathbf{x}))}{I_0(k(\mathbf{x}))}\right| ||\nabla k|| \cos (\nabla k \cdot \hat{\mathbf{v}}) \end{aligned} \end{aligned}$$

where $||\cdot ||$ denotes the $L_2$-norm and we use the fact that $||\hat{\mathbf{v}}||=1$. Therefore,

$$\begin{aligned} \begin{aligned} \left| \dfrac{\nabla q \cdot \hat{\mathbf{v}}}{q}\right| =\left| \dfrac{\left( e^{k(\mathbf{x})\,\mathbf{u}\cdot \hat{\mathbf{v}}}-e^{-k(\mathbf{x})\,\mathbf{u}\cdot \hat{\mathbf{v}}}\right) }{\left( e^{k(\mathbf{x})\,\mathbf{u}\cdot \hat{\mathbf{v}}}+e^{-k(\mathbf{x})\,\mathbf{u}\cdot \hat{\mathbf{v}}}\right) }\,(\mathbf{u}\cdot \hat{\mathbf{v}})-\,\dfrac{ I_1(k(\mathbf{x}))}{I_0(k(\mathbf{x}))}\right| ||\nabla k|| \left| \cos (\nabla k \cdot \hat{\mathbf{v}})\right| \end{aligned} \end{aligned}$$

Recalling that $|a-b|\le |a|+|b|$, $-1\le \dfrac{\left( e^{k(\mathbf{x})\,\mathbf{u}\cdot \hat{\mathbf{v}}}-e^{-k(\mathbf{x})\,\mathbf{u}\cdot \hat{\mathbf{v}}}\right) }{\left( e^{k(\mathbf{x})\,\mathbf{u}\cdot \hat{\mathbf{v}}}+e^{-k(\mathbf{x})\,\mathbf{u}\cdot \hat{\mathbf{v}}}\right) }\le 1$ and $|\cos \,(\cdot ) |\le 1$, we get

$$\begin{aligned} \left| \dfrac{\nabla q\cdot \hat{\mathbf{v}}}{q}\right| \le \left( 1+\left| \dfrac{I_1(k(\mathbf{x}))}{I_0(k(\mathbf{x}))}\right| \right) ||\nabla k||\,. \end{aligned}$$

Considering Eq. (1.12) in Laforgia and Natalini (2010) for $\nu =1$, we obtain that $\left| \dfrac{I_1}{I_0}\right| <1$, and, therefore,

$$\begin{aligned} \left| \dfrac{\nabla q\cdot \hat{\mathbf{v}}}{q}\right| < 2||\nabla k|| \end{aligned}$$

that implies

$$\begin{aligned} \max _{\mathbf{x}\in \Omega } \max _{\hat{\mathbf{v}}\in {\mathbb {S}}^{d-1}}\left| \dfrac{\nabla q\cdot \hat{\mathbf{v}}}{q}\right| < 2 \max _{\mathbf{x}\in \Omega } ||\nabla k||. \end{aligned}$$

This translates into

76 $$\begin{aligned} l_q\ge \dfrac{1}{2\max \limits _{\mathbf{x}\in \Omega }||\nabla k||}\,. \end{aligned}$$

In particular, if there exists $\mathbf{x}$ such that $\nabla k(\mathbf{x}) \cdot \hat{\mathbf{v}}=1$ and, at the same time, also satisfies $\nabla k(\mathbf{x}) \parallel \mathbf{u}$, then (76) is true with the equal sign. In particular, for the symmetry of (74) and (72) we shall consider

$$\begin{aligned} l_q\approx \dfrac{1}{2\max \limits _{\mathbf{x}\in \Omega }||\nabla k||}\,. \end{aligned}$$
